# Scalable camera traps for measuring the attractiveness of sugar baits for controlling malaria and dengue vectors

**DOI:** 10.1186/s13071-024-06539-4

**Published:** 2024-12-03

**Authors:** Felician C. Meza, Frank C. Tenywa, Simon Ashall, Fredros O. Okumu, Sarah J. Moore, Frederic Tripet

**Affiliations:** 1 Ifakara Health Institute, Environmental Health, and Ecological Science, P.O. Box 53, Ifakara, United Republic of Tanzania; 2 School of Public Health, University of the Witwatersrand, Johannesburg, South Africa; 3 School of Biodiversity, One Health and Veterinary Medicine, University of Glasgow, Glasgow, UK; 4 Centre for Applied Entomology and Parasitology, Keele University, Huxley Building, Staffordshire, UK; 5 Nelson Mandela African Institute of Science and Technology, Arusha, Tanzania; 6 Swiss Tropical & Public Health Institute, Kreuzstrasse 2, 4123 Allschwil, Switzerland; 7 University of Basel, Petersplatz 1, 4001 Basel, Switzerland

**Keywords:** ATSB, ASB, Camera trap, Malaria, Dengue, *Aedes* and *Anopheles*

## Abstract

**Background:**

Attractive targeted sugar baits (ATSBs) are promising new interventions that can complement existing vector control tools. However, reproducible and quantitative information on the level of attractiveness of ATSBs under field conditions is needed. Therefore, we customized camera traps for close-up imaging. We integrated them into a rugged ATSB monitoring station for day and nighttime recording of mosquitoes landing on the bait.

**Methods:**

The camera traps were evaluated in a semifield system and then in the field in rural Tanzania. In semifield experiments, camera traps were set up in large cages (2 m × 5 m × 2 m) to record mosquitoes landing on an attractive sugar bait (ASB), a blank ASB, or 20% sucrose (w/v). Next, 198 mosquitoes (33 males and 33 females of *Anopheles arabiensis*, *An. funestus* and *Aedes aegypti*) were released into each large cage and allowed to seek a sugar meal for 72 h with a camera recording images of the mosquitoes present on the ASB at 1-min intervals. In the field, 16 camera traps were set in 16 households, 7 with ASB attractant, 7 with ASB blank, and 2 with 20% sucrose (w/v). Human landing catch (HLC) was performed on the same nights as the camera trap recordings.

**Results:**

Under semifield conditions, significantly more mosquitoes visited the ASBs than the blank baits, with *An. funestus* visiting more frequently than *An. arabiensis*. There were no significant differences between female and male *An. arabiensis* visits, but female *An. funestus* visited more than their conspecific males did. The duration of visits did not vary between the ASB and blank controls or between the mosquito species. Moreover, mosquitoes visited the ASB or sucrose equally, with *An. arabiensis* visiting the baits more than *An. funestus*. Compared with male mosquitoes, female mosquitoes visited the baits more often. There was no significant difference in visit duration between the species.

In the field study, a mean of 70 *An. arabiensis* were caught per person per night on HLC, while 1 individual was caught per night on ASBs. There were significantly more visits by mosquitoes to the ASB than to the ASB blanks or sucrose solution, with more *An. arabiensis* visiting the baits than *An. funestus* or *Culex quinquefasciatus.* Significantly more females than males visited the baits of all the species. Again, the duration of visits was similar among *An. arabiensis*, *An. funestus* and *C. quinquefasciatus*. *Aedes aegypti* very rarely visited ASBs in the semifield experiments, and none were observed on baits in the field.

**Conclusions:**

Using camera traps to record still images of mosquitoes on ASBs offers reliable, reproducible and quantitative information on their attractiveness in various environmental conditions. Thus, camera traps serve as effective tools for evaluating and improving ATSB technology.

**Graphical abstract:**

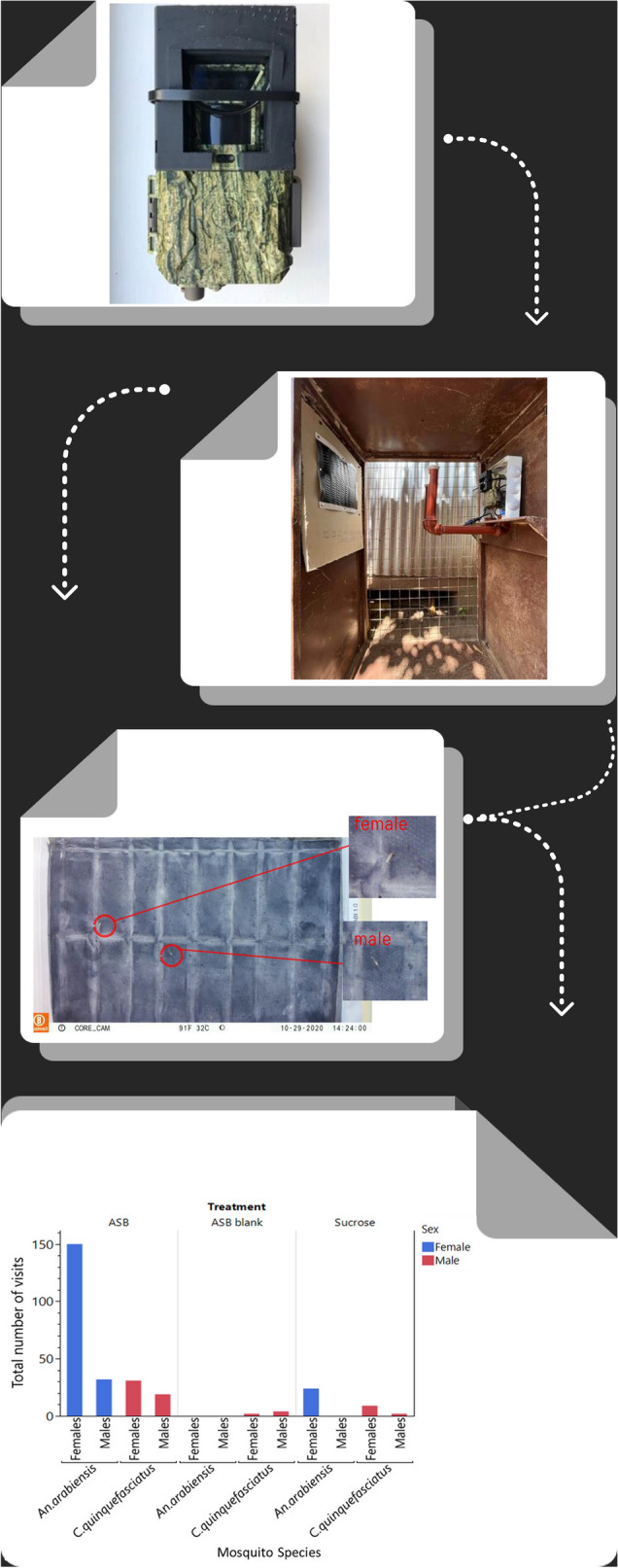

**Supplementary Information:**

The online version contains supplementary material available at 10.1186/s13071-024-06539-4.

## Background

Mosquitoes put almost half of the global population at risk of malaria and dengue. In 2022, there were an estimated 608,000 deaths and 249 million cases of malaria worldwide, with an estimated 100–400 million dengue infections occurring each year [1, 2]. Improved control of malaria and dengue vectors necessitates new vector control tools that can work synergistically with existing interventions like insecticide-treated nets (ITNs) and indoor residual spraying (IRS) [3]. Attractive targeted sugar bait (ATSB) is one example of such complementary tools and is expected to enter widespread use soon. ATSBs take advantage of mosquitoes' sugar-feeding behaviors by luring them onto a sugar-laden target treated with a lethal agent such as an insecticide [4]. Mosquitoes rely on plant sugars such as nectar and sap from flowers, leaves and plant stems as a source of their energy [5, 6] and regularly feed on plant sugars in their natural environment [7]. It is thus plausible that female mosquitoes utilize plant odors to locate host plants, and recent studies have demonstrated that *Anopheles gambiae* females can detect sesquiterpenes and alkenes derived from plants [8].

A number of ATSBs with different attractants have been developed and evaluated in the laboratory setting, semifield cages, experimental huts or field trials [9–11]. These tests have proven that ATSBs are a promising intervention, capable of complementing current vector control intervention for tackling even insecticide resistance and for outdoor or daytime biting mosquitoes in arid and semiarid areas [12–16]. In the tropics, where there is dense vegetation rich in sugar sources, ATSBs may be located near mosquito breeding habitats, outdoors and even indoors [9, 10, 17]. In this context, selecting effective attractants for ATSBs is particularly critical.

The current Sarabi ATSB stations developed by Westham Co. have a permeable membrane that encloses the bait mixture (attractant or sugar, with or without an active ingredient). A permeable membrane allows mosquitoes to feed through it and attractants to pass through it, but it is strong enough to protect the bait mixture from excessive leakage and environmental hazards such as rain, dehydration and ultraviolet light. These baits are thought to be unattractive and/or inaccessible to nontarget organisms (NTOs), such as bees and butterflies. Previous versions of these ATSBs were found to be very effective at targeting malaria vectors in a field trial in Mali [11]. Since then, a new ATSB design (version 1.2.1) has been under evaluation in Mali, Kenya and Zambia in partnership with the Innovative Vector Control Consortium (IVCC) and was included in this study, albeit without its toxic agent.

Laboratory, semifield and field trial systems are needed to evaluate the attractiveness of ATSBs to mosquitoes. Most studies assessing the attractiveness of ATSB rely on using a version of the bait without insecticide (referred to as Attractive Sugar Bait, ASB) and coloring it with food dye or including the dye in ASB sprayed on vegetation to measure the proportion of captured mosquitoes that have fed on the bait [9, 18–21]. Mosquitoes that have fed on the ASB attractant mixed with food dyes are identified by looking for the food dye in their abdomens and are scored as fed if the dye is detected. These methods require experienced personnel who may be able to detect the dye and observe the engorged abdomens of mosquitoes even when the mosquitoes have consumed small meals [22]. Some studies use a fluorescent dye rather than a food dye to identify which mosquitoes have eaten [23] or use trypan blue dye, which serves as a visual marker and is readily detected within mosquito abdomens; blue fecal spots provide additional evidence of sugar feeding [24]. In addition to requiring a fluorescence microscope and trained personnel, dyes could affect the attractiveness or palatability of bait and therefore alter the feeding rate [23], thus leading to low feeding rates and undermining the efficacy of the attractant.

Over the last decade, driven by a strong demand by hobbyists (hunters, photographers, garden owners) and professionals (nature conservationists, biologists, environmentalists and others), trail camera traps have decreased in size and gained the ability to define pictures more easily and provide HD videos. Importantly, as the camera price has decreased to < $100 per camera, due to their small size, waterproofing and low battery consumption, they have become important tools for ecologists [25, 26].

To address the need for reproducible and quantitative information on mosquito responses to lethal sugar baits, this study investigated using scalable camera trap stations to evaluate the attractiveness of ASBs under semifield and field setups. Cameras were used to record attractiveness as a key component of ASB efficacy as well as differences in the timing of visits to ASBs, duration between setting, species, mosquito sex and NTO behavior around baits. Such studies provide crucial information ahead of the larger-scale deployment of ATSBs in various regions of sub-Saharan Africa.

## Methods

### Camera trap customization

#### Camera trap customization for close-up imaging

The Bushnell dual-sensor 30-megapixel CoreDS camera (Bushnell, Cody Overland Park, KS, USA) was identified as one of the best choices for this project because it is equipped with separate day and night image sensors for improved image quality under natural daylight and nocturnal illumination through LED infrared flash units. Therefore, this camera costs slightly more than average camera traps (~ $150–200). The camera was further customized to generate close-up day and night images of the ASBs. This process was performed in three steps. First, different combinations of close-up filters and focal distances between the camera and ASB were compared to identify those with the least image distortion and best field of view. Second, the best combination was used to test different camera settings for daylight sensors and images. Finally, the best camera settings for the night sensor and images were identified, and further customizations were made to improve the night image quality. The latter required dismantling the cameras to separate the built-in Infra-Red (IR) LED flashes from the electronic circuit board and rewiring the LED IR flashes with cables and connectors so that they could be repositioned on side brackets to illuminate the baits from the sides, thereby avoiding detrimental reflections and improving contrast.

A prototype camera rig was developed and tested in a large cage in the insectaries of the Centre for Applied Entomology and Parasitology at Keele University (Additional file 1. Fig. S1). This combination of camera and close-up filtering allowed successful recording of time-lapse images of ASB baits day or night.

Further optimizations were conducted to develop a sturdy plastic adapter to hold the close-up lens in position. This was accomplished by carving a wax prototype, which was used to generate a starting STL file. The design of the adapter was improved through several rounds of printing and improvements. The resulting adapter was printed in large numbers in black plastic resin. It holds the close-up lens tightly, incorporates water- and dustproofing features and is kept in place via a plastic tie (Additional file 2. Fig. S2).

In addition, we developed simple guidelines for camera setup and operation and drafted plans to construct lockable camera stations that were both animal- and human-proof. These monitoring stations use a folded aluminum plate design with wire mesh, making them suitable for deployment in semifield and field settings (Additional file 3: Text S1, Additional file 4. Fig. S3).

#### Attractive sugar baits

For the camera optimization performed in Keele, Sarabi ATSB stations without toxic active compounds (ASBs) (version 1.0) were supplied by Westham Co. ASBs. The platform comprises a two-dimensional (20 × 28 cm) bait station with a permeable membrane on the front that encapsulates the bait mixture (attractant, sugar, with or without active ingredient), forming 16 bait pockets sealed to a flat back layer. The permeable membrane features pores that enable mosquitoes to feed through it without puncturing it, protects the bait mixture from leakage and reduces the impact of environmental hazards such as rain, dust, temperature, pressure and others (Additional file 5. Fig. S4).

### Semifield experiment setup

#### Semifield system

Semifield experimental tests were carried out inside a large screened cage in the Mosquito City facility at Ifakara Health Institute (IHI) in Kining’ina village (8.10800°S, 36.66585°E) [27–29]. Adult mosquitoes aged 3–6 days and sugar starved for 6 h were sourced from the Vector Sphere insectary at the Ifakara Health Institute and maintained at 28 ± 20 °C and 75 ± 10% humidity under a 12-h light/12-h dark cycle.

#### Camera stations for recording mosquitoes and nontargeted insects landing on ATSBs

**Fig. 1:**
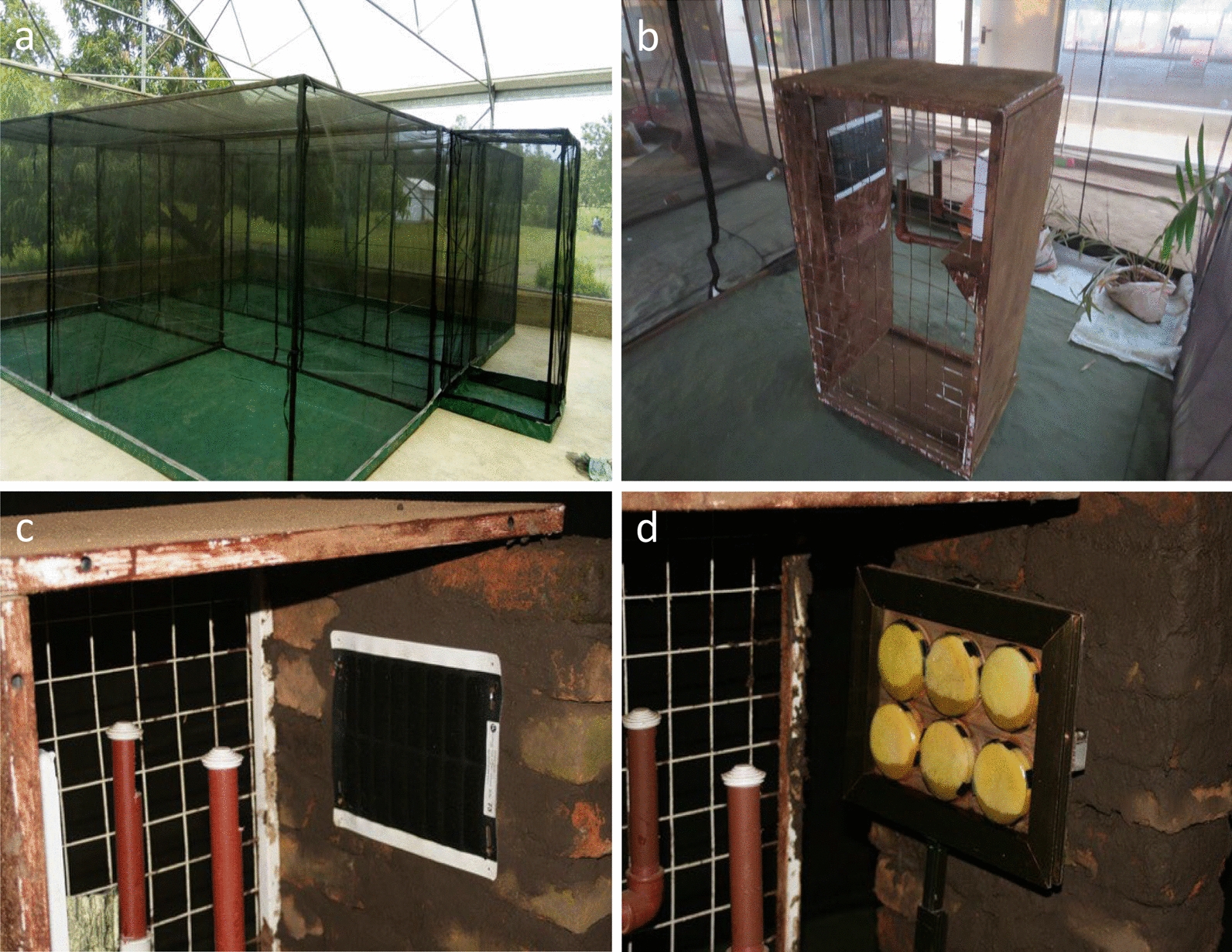
**a–d** Testing of camera stations to monitor landings on ASB with attractant, ASB blank and 20% sucrose inside the net chambers and cages: **a** A two 2 m × 5 m × 2 m cage within a semifield system, **b** potted plants added to create an environment suitable for mosquitoes, **c** ASB mounted sideways (landscape) to match the orientation of the camera's field of view, **d** 20% sucrose bait in six Petri dishes overlaid with cling film

The customized Bushnell CoreDS cameras were fitted in a 1.2 × 0.7 × 0.52-m metal frame and positioned with their objective 90 cm high, exactly opposite the center of an ASB station (Westham, Hod-Hasharon, Israel) positioned 55–60 cm away. Cameras were constructed with 3D-printed adapters and close-up filters. The ASBs were mounted sideways (landscape) to match the orientation of the camera's field of view and completely fill it (Fig. 1c, Additional file 6. Fig. S5). In addition to featuring a holding box to hold the camera in place at the correct distance, two metal brackets were welded on each side of the camera to hold the LED IR flash. The sides of the camera stations were enclosed by a metal wire frame, and the stations were locked to safeguard the camera during field use. The cameras were set to take pictures with 30-M (30-megapixel) definition at 1-min intervals day and night and were fitted with changeable 32 gigabyte memory cards (SanDisk, Sunnyvale, CA, USA). With this setup, the cameras could record images for 3 days without maintenance, battery or memory card changes.

#### Attractive sugar baits

For semifield studies, we used attractive sugar bait (ASB) version 1.2.1, which included the odor bait but not the insecticide, and the same bait stations without bait and insecticide, referred to as the 'ASB blank' for attraction comparison. This version has a molded plastic back layer with 16 wells containing bait and a flat membrane that covers the back layer and holds the bait in place. This membrane has pores that enable volatiles to escape and mosquitoes to feed on the bait.

### Semifield experiments

#### ASB versus ASB blank comparisons

The semifield experiments were performed in two 2 m × 5 m × 2 m cages within a semifield system (Fig. 1a). Potted plants were added to provide hiding places and contribute humidity, creating an environment suitable for mosquitoes (Fig. 1b). In the first treatment group, a camera station was placed centrally in the cage, and the camera was placed to monitor the ASB. In the second treatment group and large cage, the camera was placed in an ASB blank (with no odor bait) to be monitored. At the start of the experiment, 198 sugar-starved mosquitoes were maintained for 6 h (33 females and 33 males of *An. arabiensis, An. funestus* and *Ae. aegypti*) were introduced into each cage and given access to the test bait for 72 h (1800 h on day 1 to 1800 h on day 4). The camera was set to record images at 1-min intervals to record mosquitoes that had landed on the baits. At the end of the 72-h experiment, the images stored on memory cards from camera traps were transferred to a computer or hard drive for further analysis. Both live and dead mosquitoes were removed from the experimental cages using a Prokopack aspirator (John W. Hock Company, Gainesville, FL, USA) before starting another experiment or replicates. Three replicates of these experiments were conducted for individual species or mixed.

#### ASB versus sucrose comparisons

Subsequently, the attractiveness of ASB was also compared to that of 20% sucrose bait (w/v) (Fig. 1d). Sucrose baits were prepared by dissolving 20 g sucrose in 100 ml distilled water. Six Petri dishes (Sigma Aldrich), approximately 11 cm diameter and 90 ml volume, were prepared. A foam disc was prepared from a locally purchased cellulose dish washing sponge (O-Cel-O, Scotch Brite), dipped into the prepared bait solution and then pressed into each Petri dish. The Petri dishes were overlaid with one layer of cling film, and the film was pierced (10 holes) with sterile 24-mm office pins to allow the bait solution to form small droplets at each piercing point without leaking on the surface of the cling film. After six Petri dishes had been overlain with cling film and arrayed in each bait, the bait (Fig. 1d) had a surface area comparable to that of the Westham ASBs. In the first treatment group, a camera station was placed centrally in the cage, and the camera was placed to monitor the ASB. In the second treatment group and large cage, the camera was placed to monitor the sucrose bait. All the other methods used were similar to those used for the ASB vs. ASB blank comparisons. Two replicates of the ASB vs. sucrose comparison were conducted.

### Field experiments

#### Study site

The small field trial was carried out in Lupiro village (8^o^385^’^S and 36^o^670^’^E) in Ulanga District, southeastern Tanzania. The village is situated 270 m above sea level in the Kilombero River valley, 26 km south of Ifakara town, where IHI is located (Additional file 7. Fig. S6:). It is bordered by numerous small, contiguous and perennially swampy rice fields to the north and east. The area receives annual rainfall between 1200 and 1800 mm, with temperatures ranging from 20 °C to 34 °C. Throughout the year, Lupiro has high densities of *An. arabiensis*, making up > 99.9% of the *An. gambiae* complex species [30, 31]. In that region, *An. arabiensis* populations show resistance to pyrethroid insecticides (mortality < 20%). The area experiences perennial meso-endemic malaria with consistently high mosquito densities throughout the year, peaking between January and May.

#### Preliminary human landing catch survey

In Lupiro village, starting 5 days (from May 1 through May 5, 2022) before the beginning of the field ATSB experiment, a baseline survey focusing on 32 households was conducted to identify households with high mosquito densities using human landing catches (HLCs). HLCs were conducted with male volunteers seated outdoors on chairs approximately 5 to 10 m from households. They exposed their lower legs to capture mosquitoes that landed on them using a mouth aspirator [32]. The collection was performed hourly for 11 h from 19:00 to 06:00 each hour. The 16 households with the highest mosquito densities (total number of mosquitoes collected per night > 20 in all cases) were included in the ATSB attractiveness experiment. All mosquitoes collected were identified to the species level in the field using a dissection microscope and the Gillies and Coetzee identification key [33, 34].

#### ASB vs. ASB blank vs. sucrose attraction experiment

The 16 selected houses, with an average household size of 2 to 5 individuals, were built with mud or brick walls and had grass-thatched roofs. They were randomly divided into three groups for the ASB attractiveness study as follows. Seven houses were assigned camera stations monitoring ASB with attractants, seven houses received camera stations with ASB blanks, and two houses received camera stations with 20% sucrose baits. All camera types were tested over the same 22 consecutive days from May 6–May 28, 2022.

Camera stations were positioned parallel to houses and less than 1 m away (Additional file 8. Fig. S7). As in the semifield experiments, camera traps were set 60 cm away from the baits mounted in a landscape orientation ~ 90 cm from the ground. Cameras were set up to record day and night images in time-lapse mode at 1-min intervals. The batteries and memory cards were changed every 3 days to ensure that images were collected for all 22 days of the experiment. The images recorded in the camera traps on an SD card were transferred to the project computer and subsequently stored on a hard drive.

During the ASB experiment, HLCs were also tested daily to measure mosquito densities in the vicinity of the houses equipped with camera stations and ASBs but at least 10 m away from the houses and camera stations. Captured mosquitoes were stored in labeled paper cups indicating the study ID, household ID, collection time and date, replicate number and treatment type (ASB with or without attractant). In the morning, the collected mosquitoes from HLCs were transferred to the field insectary, killed in a − 20 °C freezer and sorted to the species level.

### Analyses of time-lapse imaging data from camera stations

To simplify the viewing and analysis of the time-lapse images, stacks of images equivalent to 24 h (60 × 24 images) were converted to videos using the MacOS iMovie software. This allowed fast scrolling through the images for counting and recording of the landing and departure times of mosquitoes onto and from the bait stations. Time stamps on the images helped with organizing the stacks of images and recording the exact landing and departure times. From these data, the duration of the bait station visit could also be inferred directly. Videos were scanned carefully, and the zoom function was used to detect mosquitoes or nontarget organisms at bait stations. Mosquitoes were further characterized at the species level and sexed using morphological taxonomic characters such as the shape of the antennae and maxillary palps and the shape and color of the mosquito body and wings. Nontarget organisms were examined independently by two entomologists and, except for one instance, were consistently identified at the order and family levels.

### Statistical analysis

Analysis was performed using JMP^®^ Pro 16.1.0 statistical package (SAS Institute, Inc., Cary, NC, USA). Varitions in the number of visits to baits regarding bait station treatment, species and mosquito sex were tested using the chi-square test of equal proportions. Additionally, general linear models with a negative binomial distribution were used to analyze the effects of bait station treatment, camera station (nested within treatment) and day on mosquito visits per night per trap. Post hoc pairwise comparisons were then conducted using likelihood ratio tests. Replicate effects were included in the models and reported only when significant. Continuous data such as the mean number of mosquito visits per trap per day or mean duration of visits were checked for normality and homogeneity of variance and analyzed parametrically or nonparametrically, respectively.

When conducting analyses focusing on the mean visit duration of mosquitoes to ASBs or sucrose, it was observed that occasionally mosquitoes remained on sugar sources to rest long after feeding. To prevent these observations from biasing our statistical comparisons, we performed outlier analyses on the distribution of visit durations for the semifield and field ASB vs. ASB blank experiments. Both analyses identified 12 min as the threshold duration beyond which mosquitoes were likely resting rather than sugar feeding (Additional file 9. Fig. S8). Thereafter, longer visit durations were capped at 12 min for analyses.

### Ethics approvals

All experiments were conducted with ethical approval from the Institutional Review Board of the Ifakara Health Institute (IHI/IRB/AMM/No: 24-2021) and the Medical Research Coordination Committee of the National Institute for Medical Research in Tanzania (NIMR/HQ/R.8a/Vol.IX/3777).

## Results

### Semifield experiments

#### ASB versus ASB blank

**Fig. 2:**
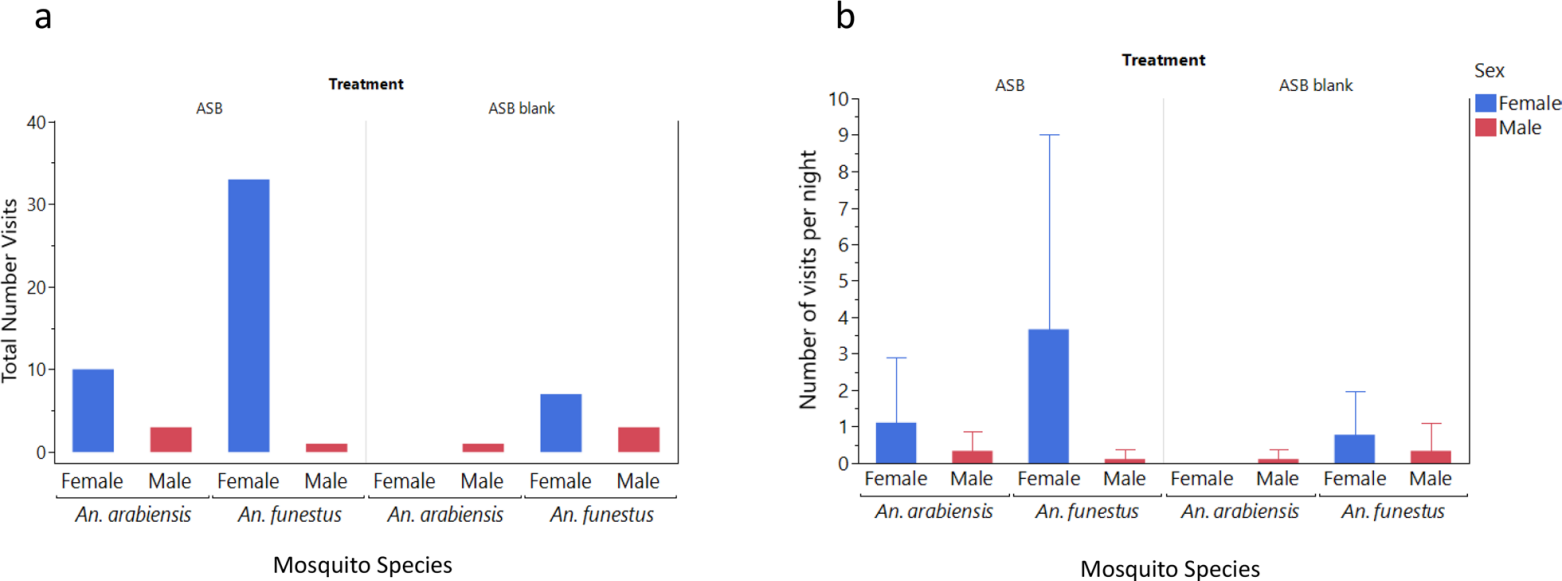
**a, b** Total number of mosquito visits **(a)** and mean number of visits per bait per night (95% confidence intervals) **(b)** to the ASB or ASB blank in semifield experiments

In semifield conditions, mosquitoes exhibited a significant preference for ASBs over ASB blanks, i.e. without attractant bait or insecticide (χ^2^ = 24.06, *P* < 0.001) (Fig. 2a, Table 1). The total number of visits was also greater for *An. funestus* on ASBs than on *An. arabiensis*, *χ2* = 16.26, *P* < 0.001 (Fig. 2a, Table 1). The female *An. arabiensis* and *An. funestus* frequented both bait types more than males did, although this difference was statistically significant only for *An. funestus* (χ^2^ = 34.18, *P* < 0.001).

**Table 1:** Total visits, mean visits per night and mean duration (min capped at 12 min) of mosquito visits to the ASB or ASB blank in the semifield experiments stratified by treatment, mosquito species and sex

Treatment	Species	Gender	Number visits	Mean visits (95%CIs)	Mean duration (95%CIs)
ASB	*Anopheles arabiensis*	Female	10	1.1 (0, 2.9)	3.5 (1.5, 5.5)
		Male	3	0.3 (0, 0.9)	4.7 (0, 20.4)
		Both	13	1.4 (0, 3.3)	3.7 (1.6, 5.9)
ASB	*An. funestus*	Female	33	3.7 (0, 9.0)	4.8 (3.6, 5.9)
		Male	1	0.1 (0, 0.4)	1.0 (NA)
		Both	32	3.8 (0, 9.1)	4.7 (3.5, 5.8)
ASB	*Aedes aegypti*	Female	0	–	–
		Male	0	–	–
		Both	0	–	–
ASB blank	*An. arabiensis*	Female	0	–	
		Male	1	0.1 (0, 0.4)	1.0 (NA)
		Both	1	0.1 (0, 0.4)	1.0 (NA)
ASB blank	*An. funestus*	Female	7	0.8 (0, 2.0)	6.1 (1.0, 11.3)
		Male	3	0.3 (0, 1.1)	1.0 (1.0, 1.0)
		Both	10	1.1 (0, 3.0)	4.6 (0.9, 8.3)
ASB blank	*Ae. aegypti*	Female	0	–	–
		Male	0	–	–
		Both	0	–	–

*NA* Not applicable, confidence intervals could not be calculated when the mean was based on a single observation

Comparisons of the mean number of visits per bait per night revealed no significant differences between ASBs and ASB blanks in attracting mosquitoes (Mann-Whitney test, *χ2* = 2.2, *P* = 0.137) (Fig. 2b, Table 1). No significant difference was observed between *An. funestus* and *An. arabiensis* (Mann-Whitney, *χ2* = 0.25*, P* = 0.6202) (Fig. 2b, Table 1). Additionally, there were no significant differences in the mean number of visits per bait per night between males and females of either mosquito species (Mann-Whitney, *χ2* = 1.27*, P* = 0.2604) (Fig. 2b, Table 1).

No significant differences were detected in the mean durations of mosquito visits between the ASB and ASB blank controls (Mann-Whitney test, χ^2^ = 1.19, *P* = 0.280) or between the *An. arabiensis* and *An. funestus* (Mann-Whitney, χ^2^ = 0.65, *P* = 0.420). However, female mosquitoes exhibited significantly longer stays on the baits than male mosquitoes did (χ^2^ = 6.8, *P* < 0.009).

**Fig. 3:**
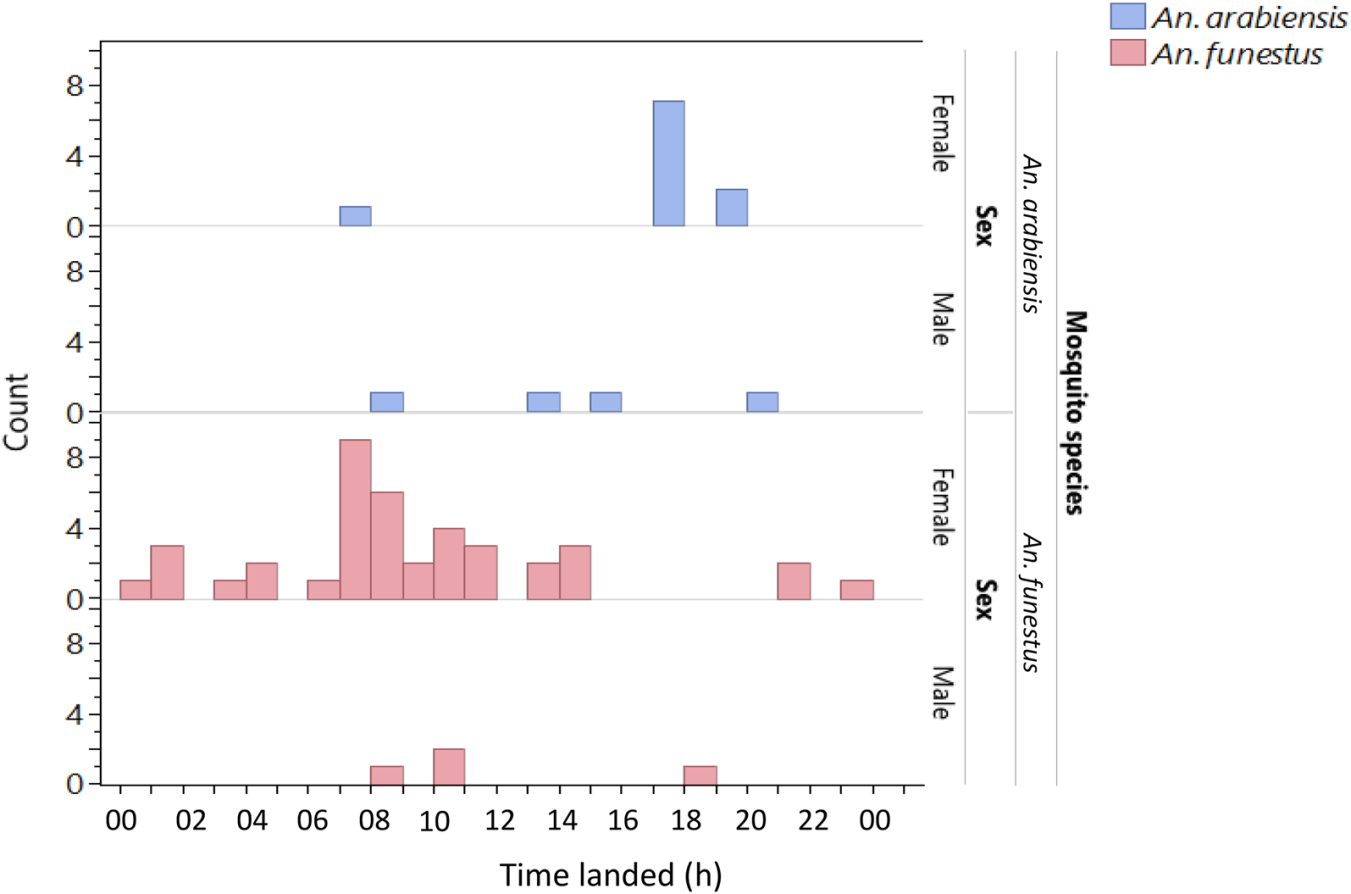
Timing of landing of mosquito species and sex on the ASB and ASB blank in the semifield cage as recorded by the camera traps

Overall, the timing of mosquito landing on the ASB and ASB blank in the semifield system ranged from 0600 to 1100 h and from 1700 to 2200 h, respectively (Fig. 3). No *Ae. aegypti* visits were recorded on baits; therefore, no analyses were conducted for that species.

#### ASB versus sucrose

**Fig. 4:**
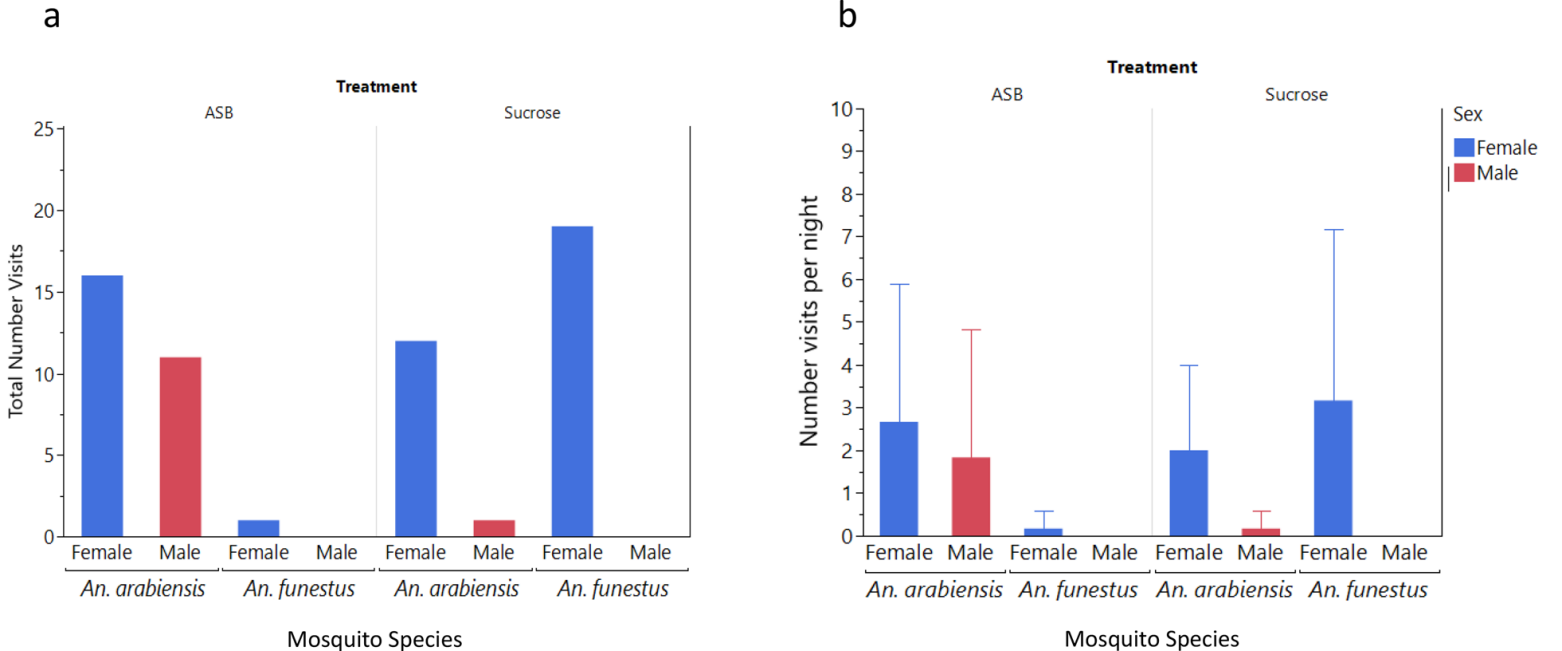
**a, b** Total number of mosquito visits **(a)** and mean number of mosquito visits per night (95% CIs) **(b)** by species and sex to the ASB or sucrose bait stations in the semifield system

According to the results of the semifield experiments, no significant difference was detected between the number of visits of ASBs and sucrose baits across the two anopheline species (chi-square test of equal proportion—likelihood ratio: χ2 = 0.27, *P* = 0.605; Table 2). Overall, *An. arabiensis* visited baits significantly more than did *An. funestus* (χ2 = 6.8, *P* < 0.009; Fig. 4a; Table 2), with *An. arabiensis* preferring ASBs (χ2 = 5.0, *P* < 0.025) and *An. funestus* favoring sucrose (χ2 = 19.8, *P* < 0.001). Females from both species visited both bait types more than males did (χ2 = 6.6, *P* = 0.010) (Fig. 4a, Table 1).

**Table 2:** Total number of visits, mean visits per night and mean visit duration (capped at 12 min) of mosquito visits to the ASB or sucrose in the semifield experiment stratified by treatment, mosquito species and sex

Treatment	Species	Gender	Number visits	Mean visits (95%CIs)	Mean duration (95%CIs)
ASB	*Anopheles arabiensis*	Female	16	2.7 (0, 5.9)	4.4 (3.4, 5.4)
		Male	11	1.8 (0, 4.8)	4.6 (3.2, 5.9)
		Both	27	4.5 (0, 10.2)	4.4 (3.7, 5.2)
ASB	*An. funestus*	Female	1	1.7 (0, 0.6)	5.0 (NA)
		Male	0	–	–
		Both	1	1.7 (0, 0.6)	5.0 (NA)
ASB	*Aedes aegypti*	Female	0	–	–
		Male	0	–	–
		Both	0	–	–
Sucrose	*An. arabiensis*	Female	12	2.0 (0.01, 4.0)	9.4 (7.0, 11.8)
		Male	1	0.2 (0, 0.6)	12.0 (NA)
		Both	13	2.2 (0.4, 4.0)	9.6 (7.4, 11.8)
Sucrose	*An. funestus*	Female	19	3.2 (0, 7.2)	8.8 (6.5, 11.2)
		Male	0	–	–
		Both	19	3.2 (0, 7.2)	8.8 (6.5, 11.2)
Sucrose	*Ae. aegypti*	Female	0	–	–
		Male	0	–	–
		Both	0	–	–

*NA* Not applicable, confidence intervals could not be calculated when the mean was based on a single observation

The mean number of visits per bait per night did not significantly differ between ASB and sucrose or between *An. funestus* and *An. arabiensis* (Fig. 4b, Table 2). Nonetheless, female mosquitoes of both species visited baits more frequently than males did (Mann-Whitney, χ2 = 6.15, *P* = 0.013) (Fig. 4b, Table 2).

Overall, there was a significantly shorter duration of mosquito visits on ASBs than on sucrose (Mann-Whitney, *χ2* = 13.98,* P* < 0.001; Table 2). No significant difference was observed in visit duration between *An. arabiensis* and *An. funestus* (Mann-Whitney, *χ2* = 3.37, *P* = 0.062), even when considering *An. arabiensis* and *An. funestus* females only (Mann-Whitney, *χ2* = 1.6, *P* = 0.200; Table 2).

**Fig. 5:**
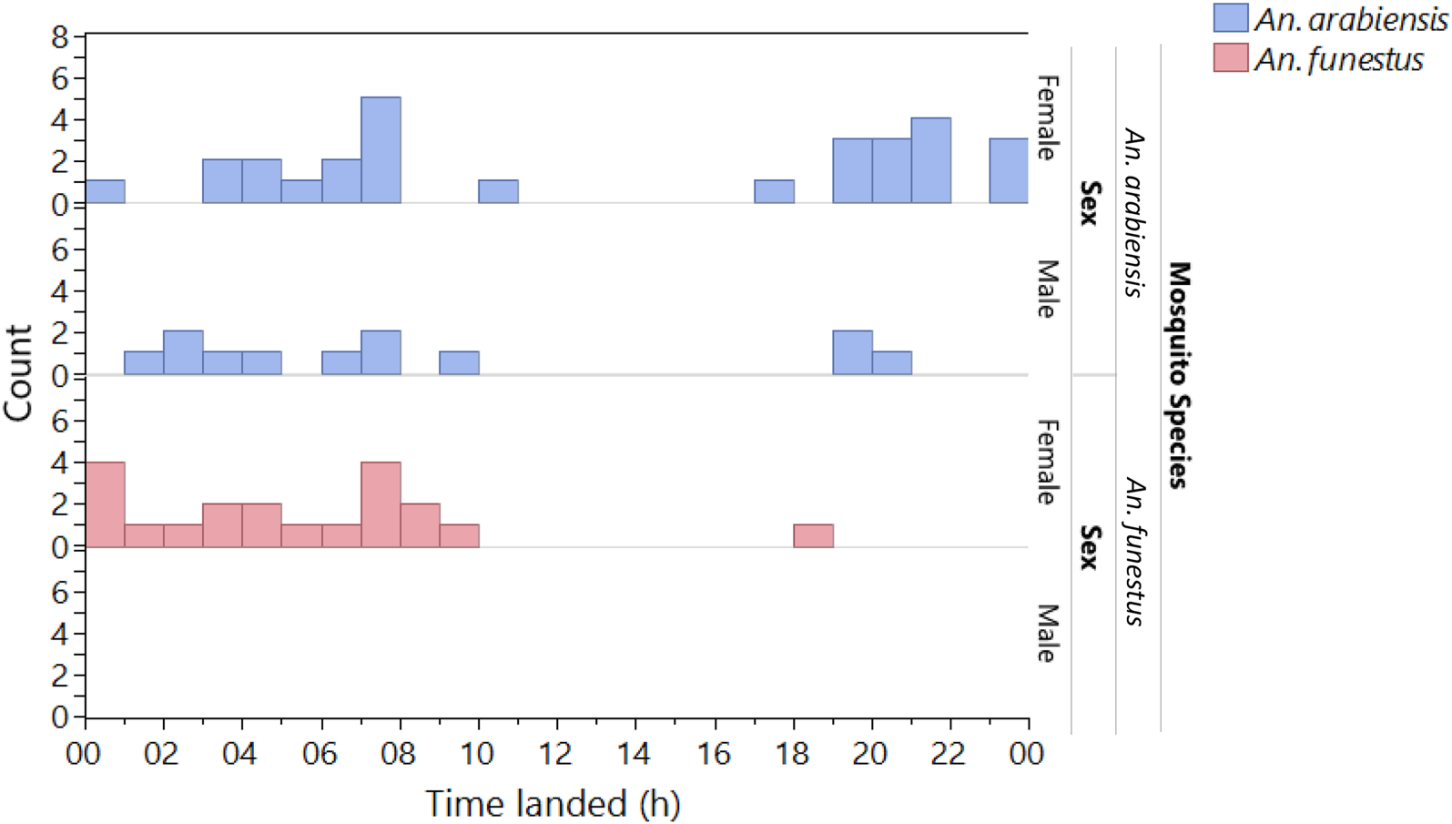
Timing of the landing of mosquito species and sex on the ASB and sucrose in semifield experiments as recorded by camera traps

Mosquitoes landed on the ASB and sucrose most frequently from 0500 to 900 h and 1700 h to 2200 h (Fig. 5). There were no visits by *Ae. aegypti*; therefore, no analyses were conducted for that species.

### Field experiment

#### Human landing catches

**Fig. 6:**
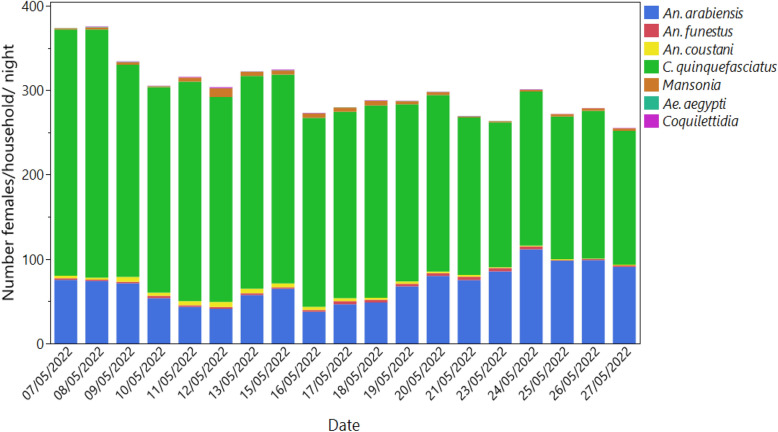
Mosquito species composition and densities described by human landing catch

The HLCs were measured on the same nights as the camera trap recordings, and the results showed that the mosquito density in Lupiro was high in May. *Culex quinquefasciatus* was the most common species that landed on capturers (223 ± 81 SD) per night per person, and *An. arabiensis* was the next most common species, landing (60.8 ± 32 SD) per night per person (Fig. 6).

**Fig. 7:**
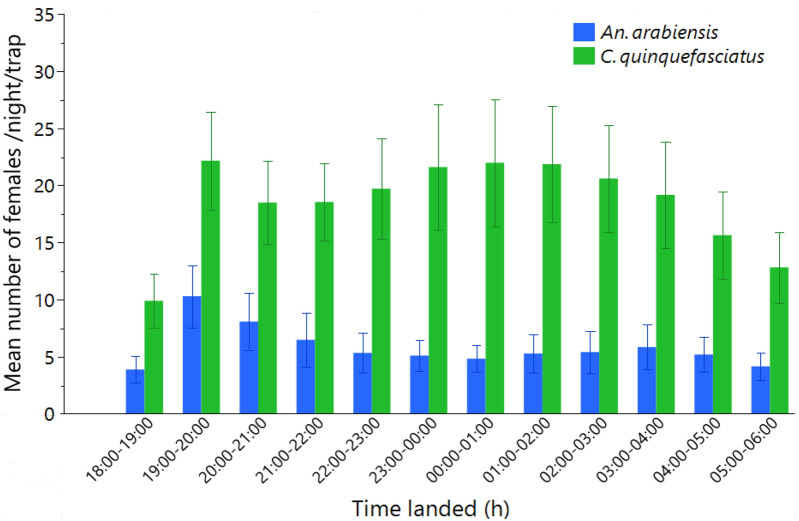
Mean number of female mosquitoes caught by HLC on the same nights as the camera trap assessments, showing the landing time at which the mosquitoes were caught. Error bars represent the standard deviation from the mean (SD)

For *An. arabiensis*, HLC landings occurred during all hours between 1800 and 0600 h but were most frequent between 1900 and 0000 h (Fig. 7). The number of landings by *C. quinquefasciatus* was much greater than that by other species and was particularly frequent between 1900 and 0400 h, after which the number of landings decreased (Fig. 7).

#### ASBs vs. ASB blanks vs. sucrose

**Fig. 8:**
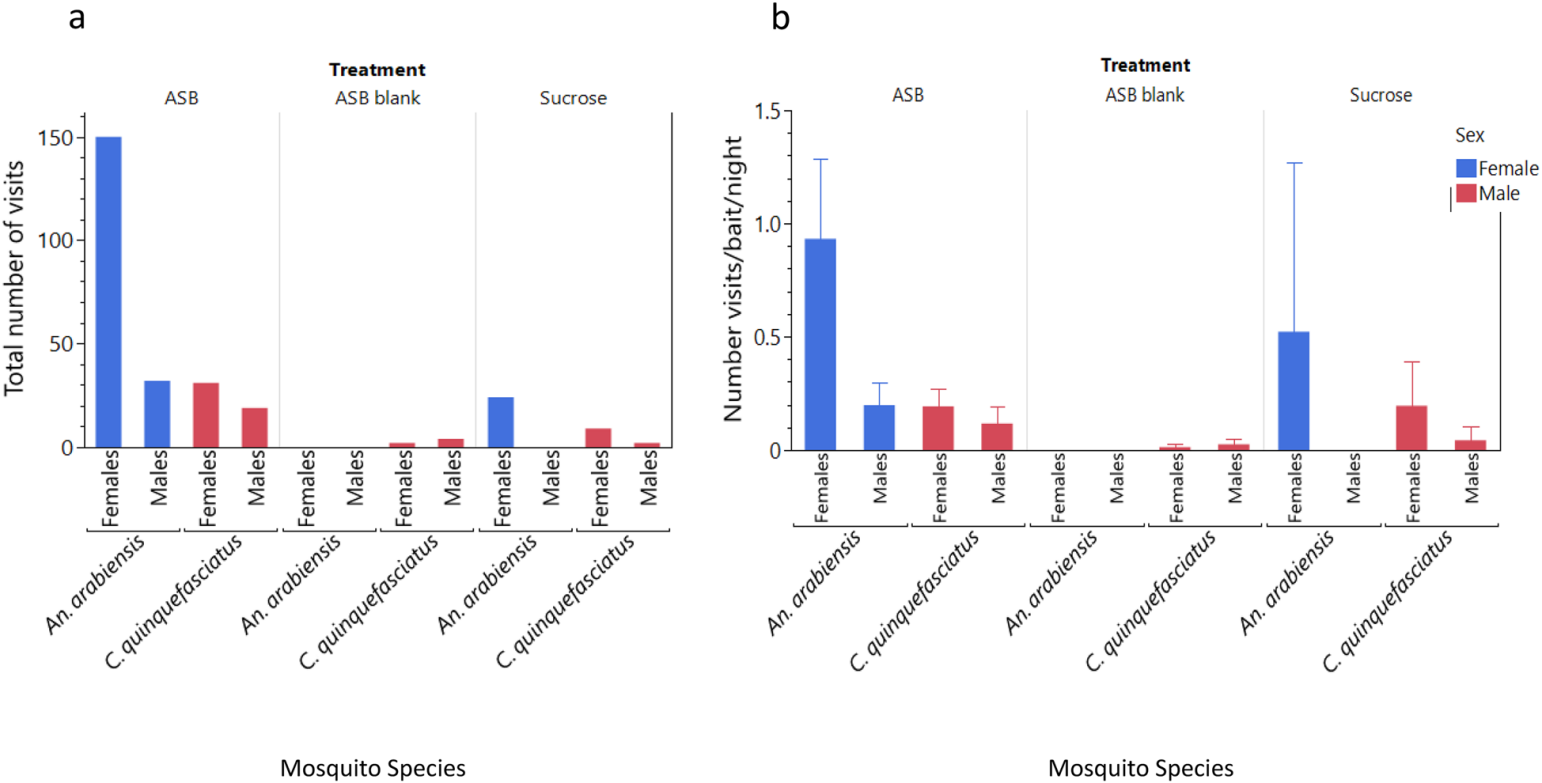
**a, b** Total number of mosquito visits by species and sex (**a**) and mean number of mosquito visits per bait per night (95% CIs) (**b**) to the ASB, ASB blank or sucrose bait in the field

In the field study, a total of 239 mosquitoes visited ASBs, while only 6 mosquitoes visited the ASB blanks (χ2 = 283.28, *P* < 0.001; Fig. 8a, Table 3). *Anopheles arabiensis* led with 182 visits, followed by 56 *C. quinquefasciatus* and 7 *An. funestus*, and there was a significant difference in the visitation rate (χ2 = 188.51, *P* < 0.001; Table 3). Female mosquitoes of *An. arabiensis*, *An. funestus* and *C. quinquefasciatus* visited baits significantly more often than males did (χ2 > 4.35, *P* < 0.037 in all cases; Fig. 8b, Table 3).

**Table 3:** Total number of visits, mean visits per bait and night and mean duration (min capped at 12 min) of mosquito visits to the ASB, ASB blank or sucrose bait stations in the field experiment broken down by treatment, mosquito species and sex

Treatment	Species	Gender	Number visits	Mean visits (95%CIs)	Mean duration (95%CIs)
ASB	*Anopheles arabiensis*	Female	150	0.9 (0.6, 1.3)	4.6 (4.1, 5.1)
		Male	32	0.2 (0.1, 0.3)	2.3 (1.3, 3.0)
		Both	182	1.1 (0.7, 1.5)	4.2 (3.7, 4.7)
ASB	*An. funestus*	Female	7	0.04 (0.01, 0.08)	3.6 (0.4, 6.8)
		Male	0	–	–
		Both	7	0.04 (0.01, 0.08)	3.6 (0.4, 6.8)
ASB	*Culex quinquefasciatus*	Female	31	0.2 (0.1, 0.3)	4.7 (3.1, 6.3)
		Male	19	0.1 (0.05, 0.2)	3.0 (1.4, 4.5)
		Both	50	0.3 (0.2, 0.4)	4.1 (3.0, 5.2)
ASB blank	*An. arabiensis*	Female	0	-	-
		Male	0	-	-
		Both	0	-	-
ASB blank	*An. funestus*	Female	0	-	-
		Male	0	-	-
		Both	0	-	-
ASB blank	*C. quinquefasciatus*	Female	2	0.01 (0, 0.03)	8.0 (0, 58.8)
		Male	4	0.02 (0, 0.05)	2.6 (0, 6.0)
		Both	6	0.04 (0, 0.07)	4.5 (0.3, 8.7)
Sucrose	*An. arabiensis*	Female	24	0.5 (0, 1.3)	6.9 (5.1, 8.6)
		Male	0	-	-
		Both	24	0.5 (0, 1.3)	6.9 (5.1, 8.6)
Sucrose	*An. funestus*	Female	0	–	–
		Male	0		–
		Both	0		–
Sucrose	*C. quinquefasciatus*	Female	9	0.2 (0, 0.4)	10 (7.4, 12.6)
		Male	2	0.04 (0, 0.1)	2.0 (0, 14.7)
		Both	11	0.2 (0, 0.4)	8.6 (5.6, 11.5)

There was a significant difference in visit duration among ASB, ASB blanks and sucrose bait according to the Kruskal-Wallis test (*χ2* = 16.26, *P* < 0.001; Table 3), with mosquitoes spending more time on sucrose compared to ASBs (Dunn test: *Z* = 4.02, *P* < 0.001). However, they did not spend more time on sucrose bait compared to ASB blanks (Dunn test: *Z* = 1.29, *P* = 0.587). Additionally, no significant difference was found in visit duration among *An. funestus*, *An. arabiensis* and *C. quinquefasciatus* (Mann-Whitney*, χ2* = 0.63, *P* = 0.731; Table 3). No *Ae. aegypti* visited any baits in the field study.

**Fig. 9:**
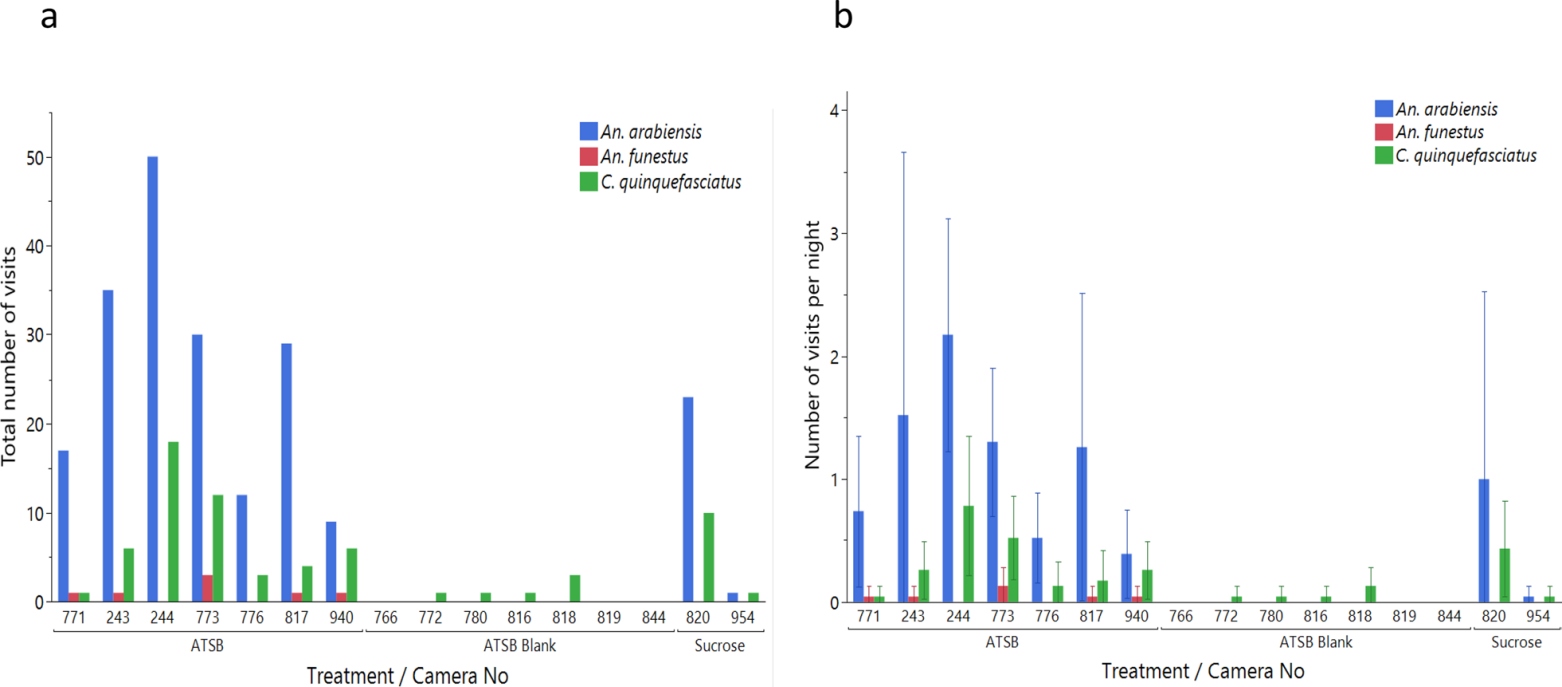
**a, b** Total number of mosquito visits per camera trap (**a**) and mean number of mosquito visits per camera trap per night per treatment (**b**) in each treatment in the field

The mean number of nightly visits per trap differed between treatment groups and followed the same overall pattern as that observed for the total number of visits (Kruskal-Wallis test: *χ2* = 130.40, *P* < 0.001) (Fig. 9). The overall frequency of mosquito visits per trap-night was the highest for ASBs, followed by a significant decrease in the frequency of mosquito visits per trap-night for sucrose and further decrease in the frequency of mosquito visits per trap-night for ASB blanks (n test: Z > 2.75, *P* < 0.018 for both).

Using the data from the ASB and ASB blanks for which the number of bait stations used was equal, we also fitted the number of visits by *An. arabiensis* and *C. quinquefasciatus* into two nested general linear models to account for variation between camera stations (Table 4). The models confirmed the very significant impacts of treatment, camera station (nested within treatment) and date on the frequency of visits of both species (Fig. 9 and Table 4). The female *An. arabiensis* visited baits more frequently than males did, but no significant difference between sexes was observed for *C. quinquefasciatus* (Table 4).

**Table 4:** General linear models of the effect of treatment (ASB with or without attractant), mosquito sex, camera station and date on the mean number of visits per night to baits by *Anopheles arabiensis* and *Culex quinquefasciatus*

Species	Effect tests	*df*	χ2	*P value*
*An. arabiensis*	Treatment	1	197.33	< 0.001 ***
	Sex	1	83.04	< 0.001***
	Camera station [treatment]	12	49.02	< 0.001***
	Date	22	96.65	< 0.001***
*C. quinquefasciatus*	Treatment	1	20.93	< 0.001***
	Sex	1	1.80	0.180 NS
	Camera station [treatment]	12	36.21	< 0.001***
	Date	22	54.91	< 0.001***

^***^P < 0.001

*df* degrees of freedom, *NS* no significant difference

**Fig. 10:**
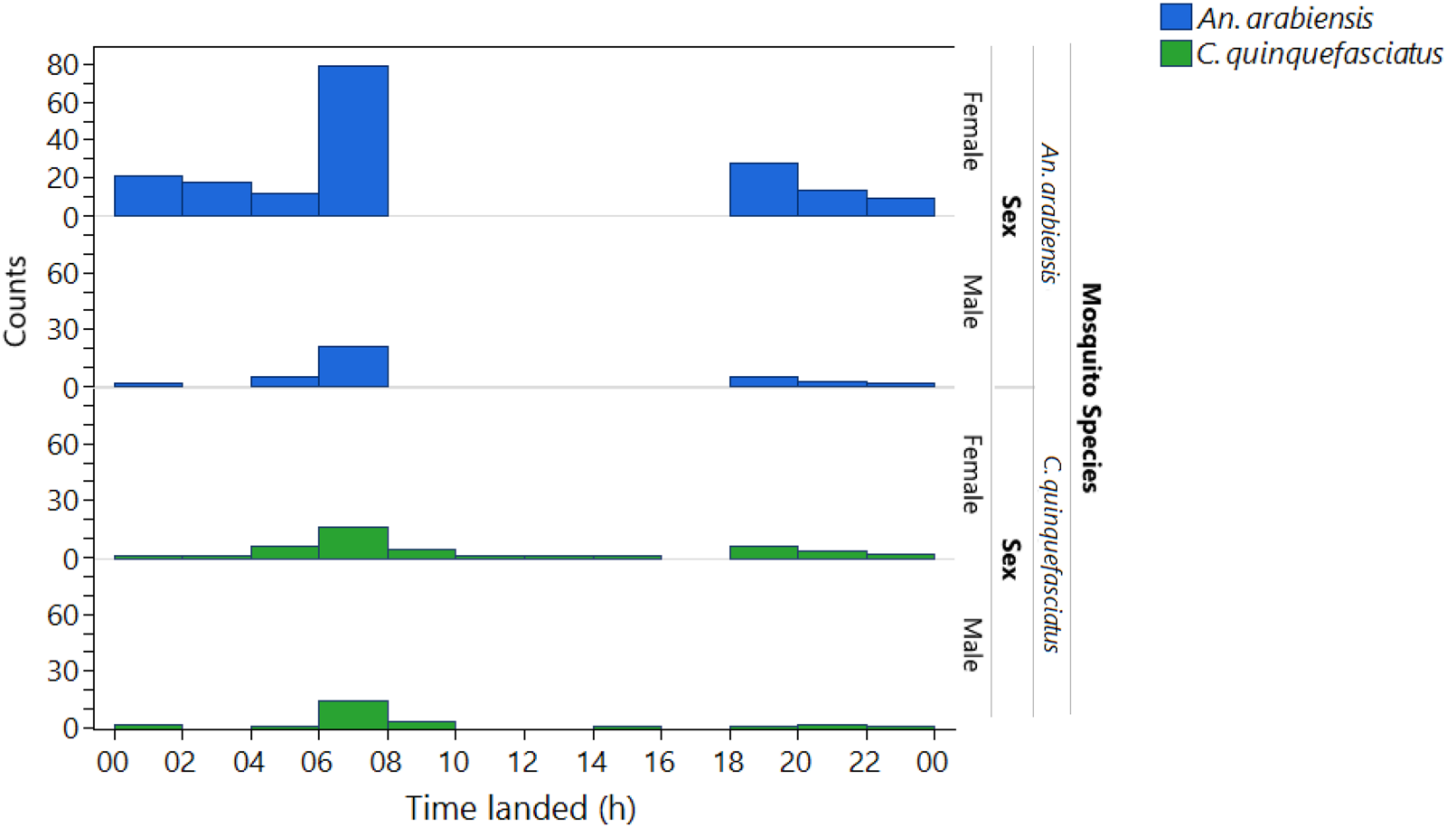
Time spent on total landings on ASBs (with and without bait) at camera stations in the field stratified by mosquito species and sex

Females and males of both species exhibited similar patterns of nocturnal visits to ASBs. Camera stations recorded most of the landings between 0500 and 0700 h, and another but lesser peak of activity occurred from 1700 to 1900 h (Fig. 10). Very few visits took place in the daytime, and these were made only by *C. quinquefasciatus.*

Camera stations also recorded nontarget organisms (NTOs) visiting ASBs. These were uncommon and were detected on only 32 of the total 423,360 images analyzed in this study (0.0075%). NTOs were found on only three of the ASB stations and never on any of the ASB blanks or the sucrose baits. The NTO strains detected were ants, spiders, moths, wasps and cockroaches. Among all the camera traps, camera number 817 was visited more often by NTOs than by the other camera traps (Table 5).

**Table 5:** List of image files on which nontarget organism species (NTOs) were recorded; these were identified as order and family

Image file	Treatment	Camera number	Date	Time	Night/day	Group	Order	Family	Common name
5080027	ASB	817	08/05/2022	05:02	Night	Invertebrate	Hymenoptera	Formicidae	Ant
5080028	ASB	817	08/05/2022	05:03	Night	Invertebrate	Hymenoptera	Formicidae	Ant
5080038	ASB	817	08/05/2022	05:13	Night	Invertebrate	Hymenoptera	Formicidae	Ant
5080068	ASB	817	08/05/2022	05:43	Night	Invertebrate	Hymenoptera	Formicidae	Ant
5080069	ASB	817	08/05/2022	05:44	Night	Invertebrate	Hymenoptera	Formicidae	Ant
5080242	ASB	817	08/05/2022	08:38	Day	Invertebrate	Aranaea		Spider
5080244	ASB	817	08/05/2022	08:39	Day	Invertebrate	Aranaea		Spider
5120035	ASB	773	12/05/2022	03:19	Night	Invertebrate	Lepidoptera		Moth
5120043	ASB	773	12/05/2022	03:27	Night	Invertebrate	Lepidoptera		Moth
5140190	ASB	817	14/05/2022	13:49	Day	Invertebrate	Hymenoptera	Vespidae	Wasp
5140191	ASB	817	14/05/2022	13:50	Day	Invertebrate	Hymenoptera	Vespidae	Wasp
5140452	ASB	817	14/05/2022	01:32	Night	Invertebrate	Hymenoptera	Formicidae	Ant
5140504	ASB	817	14/05/2022	19:04	Night	Invertebrate	Hymenoptera	Formicidae	Ant
5140598	ASB	817	14/05/2022	03:58	Night	Invertebrate	Blattodea	Blattidae	Cockroach
5140704	ASB	817	14/05/2022	22:23	Night	Invertebrate	Hymenoptera	Formicidae	Ant
5150037	ASB	771	15/05/2022	04:02	Night	Invertebrate	Hymenoptera	Formicidae	Ant
5150073	ASB	771	15/05/2022	04:38	Night	Invertebrate	Hymenoptera	Formicidae	Ant
5150074	ASB	771	15/05/2022	04:39	Night	Invertebrate	Hymenoptera	Formicidae	Ant
5150076	ASB	771	15/05/2022	04:41	Night	Invertebrate	Hymenoptera	Formicidae	Ant
5150127	ASB	771	15/05/2022	05:32	Night	Invertebrate	Hymenoptera	Formicidae	Ant
5150828	ASB	817	15/05/2022	00:27	Night	Vertebrate	Squamata	Gekkonidae	House gecko
5170358	ASB	817	17/05/2022	10:16	Day	Invertebrate	Diptera	Psychodidae	Moth fly
5170359	ASB	817	17/05/2022	10:17	Day	Invertebrate	Diptera	Psychodidae	Moth fly
5170497	ASB	771	17/05/2022	12:58	Day	Invertebrate	Hymenoptera	Vespidae	Wasp
5180032	ASB	817	18/05/2022	14:06	Day	Invertebrate	Hemiptera	Aphididae	Aphid
5140033	ASB	771	18/05/2022	14:31	Day	Invertebrate	Aranaea		Spider
5180034	ASB	771	18/05/2022	14:32	Day	Invertebrate	Aranaea		Spider
5200244	ASB	773	20/05/2022	07:22	Day	Invertebrate	Aranaea		Spider
5200271	ASB	817	20/05/2022	16:26	Day	Invertebrate	Aranaea		Spider
5230036	ASB	817	23/05/2022	07:10	Day	Invertebrate	Aranaea		Spider
5230037	ASB	817	23/05/2022	07:11	Day	Invertebrate	Aranaea		Spider
5230038	ASB	817	23/05/2022	07:12	Day	Invertebrate	Aranaea		Spider

## Discussion

The present study evaluated the attractiveness of ASB Sarabi, version 1.2.1, developed by Westham Co., for mosquito vectors in a region of south-central Tanzania with both malaria and dengue transmission using a camera station. This is the first semifield and field study to demonstrate that camera stations offer a simple solution for assessing and comparing the attractiveness of ASBs and other potential mosquito attractants. Under the semifield system, comparisons between Westham ASB Sarabi version 1.2.1 and sucrose solution showed that mosquitoes overall were similarly attracted to both. This finding is in line with a previous study conducted in coastal Tanzania in which locally made ASBs were found to be equally attractive to sucrose solution [22]. Here, however, under the semifield system, *An. arabiensis* visited the ASBs more than they did in the sucrose solution, whereas *An. funestus* did not. Comparisons between ASBs and ASB blanks lacking the attractive odor blend in the semifield system also showed that the ASB attractant was attractive to mosquitoes. The relatively low number of mosquito visits observed compared to the number released in these experiments may be attributed to the fact that they were carried out during the dry season, which results in low overall mosquito activity [35–38]. Fewer visits also meant lower statistical power; thus, seasonality and patterns of mosquito activity play major roles in the success and need for replication of such experiments.

In contrast, field studies were conducted during the rainy season and confirmed that the ASB attractant, which was used at Westham ASB Station version 1.2.1, is more effective at attracting mosquitoes than the ASB blank in the control arm. This confirms that the attraction of the Westham ASB station is associated with its odor bait and therefore that olfactory attraction appears more important than any visual attraction of the bait station. Notably, despite the clear attraction of *An. arabiensis* and *C. quinquefasciatus* to ASBs, the results obtained from the human landing catch conducted simultaneously with the camera recordings suggest a relatively low overall attractiveness of these ASB version 1.2.1 stations compared to that of humans. The HLCs indicated that mosquito densities in Lupiro village were high during the month of May, which aligns with the rainy season in the region. On average, 378 mosquitoes landed on capturers per night but an average of 2 landings on baits per night. Our findings suggest two hypotheses regarding the observed low visitation to the baits compared to the HLC. First, it may be possible that sugar feeding on the ASB bait stations was limited in the rainy period given the abundance of flowers and fruits that mosquitoes could rely on for sugar at that time. In such circumstances, mosquitoes may feed mainly on natural sugar sources because of their abundance compared to the limited number of ASBs. Therefore, this first hypothesis focuses on sugar usage as a dietary complement for energy required for flight, body maintenance and mating. A slightly different explanation is that the high availability of water sources reduces the reliance on nectar, which can be sought by mosquitoes for both its water and sugar content. During the rainy period, mosquitoes might reduce their reliance on nectar or sugar solution because they find water droplets or puddles very easily and thus exhibit an overall reduced attraction to natural sugar sources and artificial baits. Thus, in the future, it will be important to understand the dynamics of sugar feeding in relation to its dual role as a source of water and/or energy and its changes in availability throughout different seasons. Interestingly, the data collected from the two stations baited with sucrose suggest that the ASB attractant performed better than the other agents in the field, which contrasts with the findings in the semifield system. However, there were only two sucrose stations available for field comparison, so further work is needed to confirm these findings. Further work using larger sample sizes should also demonstrate whether ASB competes well in terms of long-range attraction with natural sources of sugar and nectar and under what conditions. Therefore, further studies should formally test the efficacy of ASB attractants across different seasons and geographical sites to highlight the relationship between natural sugar availability and ASB efficacy.

Regarding the specificity of the ASB attractant, this study confirmed that the Westham ASB attractant was more attractive to *An. arabiensis* than to *An. funestus* in the study area. However, this has to be tested in the semifield to determine whether it is related to relative survival or ASB attractiveness. *Anopheles arabiensis* made longer visits to the ASB station with the attractant and sucrose bait system than on the ASB blank, which is a good indication that feeding activities took place. However, *An. arabiensis* were more frequently found on ASBs in the field. Notably, this species was generally more abundant than *An. funestus* according to the HLCs. Therefore, the greater presence of *An. arabiensis* on ASBs is not necessarily indicative of greater attraction to these baits. Additionally, in the Zambia field trial [39], a greater proportion of *An. funestus* was observed to have fed on ASBs than *An. arabiensis*. These results underscore the importance of considering species-specific feeding behaviors and abundances when evaluating the efficacy and attractiveness of control measures such as ASBs.

Although the baits tested here did not include a killing agent, it is important that mosquitoes not only explore the baits but also feed on them effectively to pick up a sufficient dose of killing agent [22]. *Culex quinquefasciatus* also fed on ASBs, but their numbers on baits were much lower than expected given their great abundance in HLC catches. *Aedes aegypti* was never observed on the baits in the field despite being present in HLC catches, although at very low numbers. Under semifield settings, this species was very rarely observed on the baits. This finding suggested that *Ae. aegypti* might require a different blend of attractants or may be even more prone to feeding on natural sugar sources than *Anopheles* or *Culex*. Additionally, this study highlighted other important mosquito behavioral factors, such as sex-specific differences in attraction to sugar feeding. In our study, female mosquitoes visited the ASB more than male mosquitoes did, which may imply that the ASB attractant or bait format is more attractive to female mosquitoes but less attractive to males, although semifield sex comparisons may have been affected by the relative survival rate. In contrast to our findings, a field study in which ASB station version 1.1.1 developed by Westham Co. in Zambia was used revealed a greater proportion of uranine-positive male mosquitoes than females, implying that male mosquitoes feed more on bait stations than females [39]. Our finding of female attraction to ATSBs in south-central Tanzania supports the potential use of ASBs for malaria control programs in that region, as female mosquitoes are responsible for blood feeding on hosts, hence transmitting pathogens [40]. Interestingly, for the first time to our knowledge and through direct observation, the present study documented the timing of landing on the baits in the field by different species. In the field, landing on the ASB started at 1700 h and continued until 2000 h in the evening, with a second peak of landings occurring at approximately 0500 h to 0700 h. Therefore, the start and end times of sugar feeding were comparable to those observed in host-seeking female *C. quinquefasciatus* and *An. arabiensis* from the HLC samples performed in this study, which also aligns with the findings of studies describing the natural host-seeking behavior of *An. arabiensis, An. gambiae* s.l. and *An. funestus* [41, 42]. However, unlike for host seeking, our results and those of other studies indicate that sugar feeding typically begins early in the evening and continues for 3–4 h followed by a clear drop in activity later at night and another distinct peak of activity early in the morning [41–43].

To assess the proportion of male and female mosquitoes sugar feeding ahead of ATSB trials, other studies have used the cold anthrone method to detect sugar uptake in anopheline species [44]. Another approach used consisted of collecting the contents of light traps baited with flowers at 1-h intervals to infer the feeding timing on natural sugar by male and female *An. gambiae* s.l. [21]. Notably, such indirect methods cannot possibly generate data on the relative proportion and timing of visits to baits by vector species as accurately as those measured from direct visual recording on the ASB, as implemented in this study.

The camera stations deployed in the field also generated important data on visits by nontarget organisms (NTOs) to the baits. These visits were rare, and the taxa involved included Araneae (spiders), Hymenoptera (ants, wasps) and Lepidoptera (moths). These observations align with findings reported in previous studies [45–49]. The latter studies relied on identifying NTOs that fed from ATSB through the detection of food dye or staining in all insects collected by UV light traps, Malaise traps, plate traps, sweep nets and pitfall traps [45–49]. The varying efficacy of the trapping and marking methods used and the complexity of detection in these studies make them susceptible to various biases. Camera stations are a much more direct method for recording the attraction to baits of any NTO taxon, including visits by vertebrates such as that of a gecko, recorded in our study. We also found variation in local NTO abundance between ASBs, with camera number 817 recording more NTO visits than other camera traps. This may be attributed to the location of the camera trap at the fringe of the village in a wooded area.

No serious issues with the camera stations were observed. The camera traps produced adequate image quality with no major difficulties in recognizing species or determining the sex of mosquitoes. No major data collection difficulties were encountered, except that identifying images positive for mosquitoes or NTOs from all generated images took time. Notably, cold-blooded organisms cannot trigger the camera's built-in passive infrared trigger when landing on the ATSB or entering the camera's field of view. Therefore, we recommend using a 24-h time-lapse approach with images taken at 1-min intervals for tracking mosquito landings in ATSB studies. The downside of that approach is that one camera trap will produce approximately 4320 images per 72 h. To save time in viewing all those images, we converted 24-h stacks of images into mp4 video files using Mac iMovie software. This enabled fast scrolling through the video created to quickly detect those frames with mosquitoes and record their landing and departure times. The possibility of analyzing videos using more elaborate image analysis software assisted by machine learning is currently being evaluated [50, 51].

One important finding from our dry season semifield experiments comparing ASBs to ASB blanks was that when conditions in enclosures were very dry (> 35 °C), mosquitoes did not engage at all in our experiments and hid and died. Considering that 198 mosquitoes were released into the experimental cage and that observations were made for 72 h, we collected very few positive images of mosquitoes landing on the ASB. Thus, mosquitoes seemed to have hidden in the clay pots to mitigate desiccation but did not visit the ASB attractant under those conditions. Considering these observations, predicting what level of ASB visitation will be detected under field conditions during very dry weather is difficult. While increased sugar feeding is expected as long as mosquitoes are active, very harsh conditions are also likely to induce further protective behavior, such as actively avoiding open and arid locations, thus enabling them to conserve moisture and survive. This might include seeking shelter, resting in cool and shaded areas, or even stimulating [52, 53].

In addition to threshold levels of drought tolerance, the availability and quality of sugar sources may also play a role in mosquito tolerance. In the dry season, natural sugar sources such as nectar-producing plants might be scarce, further increasing the attractiveness of sugar-based attractants such as those used in ASBs to mosquitoes. This is supported by a study conducted in central Tanzania using semi-field enclosures, where settings with dense vegetation, sparse vegetation and no vegetation were simulated during both the dry and wet seasons [54]. Although that study was performed in a semifield with a controlled environment in both the dry and rainy seasons, it further emphasizes the need for field studies comparing ATSB attractiveness in settings or regions with dense vegetation and rich flowering plants and in regions with sparse or semiarid areas to understand the full complexity of the sugar feeding behavior of mosquitoes in the field across the season.

The camera traps used in this study are well suited for this purpose because they can generate direct data for comparisons of ATSB attractiveness in different settings. In the field setting, it may be difficult to rely on detecting food dyes [11] or uranine markers (the Westham ASB used in this study had a uranine marker, while the ASB blank had no marker) to estimate the percentage number of mosquitoes that have fed on the ASB under investigation [39]. The recapture rate in the field is normally very low because of the harsh environmental conditions [55]. It is therefore difficult to trace or capture a mosquito that visited and fed on the ASB bait station without mass ASB deployment. Additionally, the trap position seems to be critical in assessing the feeding rate when using a bait dye in the field (uranine or food dye). A study conducted in Mali, West Africa, successfully used a glue trap method to evaluate the relative attractiveness of ATSBs in the field; however, the smell of glue itself can have a repellent effect [21]. Here, again, the camera trap method can be used to assess the attraction of ATSBs without the need to deploy large numbers of ATSBs, positioning glue or other traps to indirectly evaluate ATSB attractiveness in the field. Camera stations allow simple measurements of attraction, whereas trap-based feeding rate assessments, though important, may further depend on (i) the short-range stimulus needed to feed once mosquitoes have landed, (ii) the accessibility of the bait and (iii) its palatability. Therefore, the application of camera traps in the field may arguably generate the best measure of ATSB attractiveness independent of any other factor. However, further studies may be necessary to understand whether all the landings observed led to effective feeding. This could simply be established through comparing landing rates estimated by camera stations to feeding rates estimated via dye detection in semifield studies.

## Conclusions

Using camera traps to record still images of mosquitoes present on ASBs offers reliable, reproducible and quantitative data on their attractiveness in various environmental conditions. Therefore, modified camera traps are effective tools for assessing mosquito interactions with sugar baits or other attractants in semifield and field conditions. The present study demonstrated that the ASB attractant used in the ASB stations, version 1.2.1 by Westham, is attractive to *An. arabiensis* under both semifield and field conditions while rarely attracting NTOs. Future studies using the same camera stations could help clarify the complex relationships among seasonality, rainfall, drought and sugar source availability and their impacts on absolute and relative ATSB attractiveness.

## Supplementary Information

Additional file 1.

## Data Availability

No datasets were generated or analysed during the current study.

## Abbreviations

IRS: Indoor residual spraying
ATSB: Attractive targeted sugar bait
IHI: Ifakara Health Institute
IRB: Institutional review board
ITNs: Insecticide-treated bed nets
NIMR: National Institute for Medical Research
HLC: Human landing catch
