# Supplementary material for: Scalable camera traps for measuring the attractiveness of sugar baits for controlling malaria and dengue vectors
Source: Parasit Vectors. 2024 Dec 3;17:499. doi: 10.1186/s13071-024-06539-4 (PMC11616269; doi:10.1186/s13071-024-06539-4)
Supplement: Supplementary file 1 — Additional file 1. [file 13071_2024_6539_MOESM1_ESM.docx]

**Supplementary Information Files**

**Additional file 1. Fig. S1:** The prototype camera rig used in the Keele insectaries. The two LED flash-units built in the camera were rewired and repositioned on side brackets. The close-up filter was originally held in place with rubber bands and a piece of Styrofoam (red arrow).

**
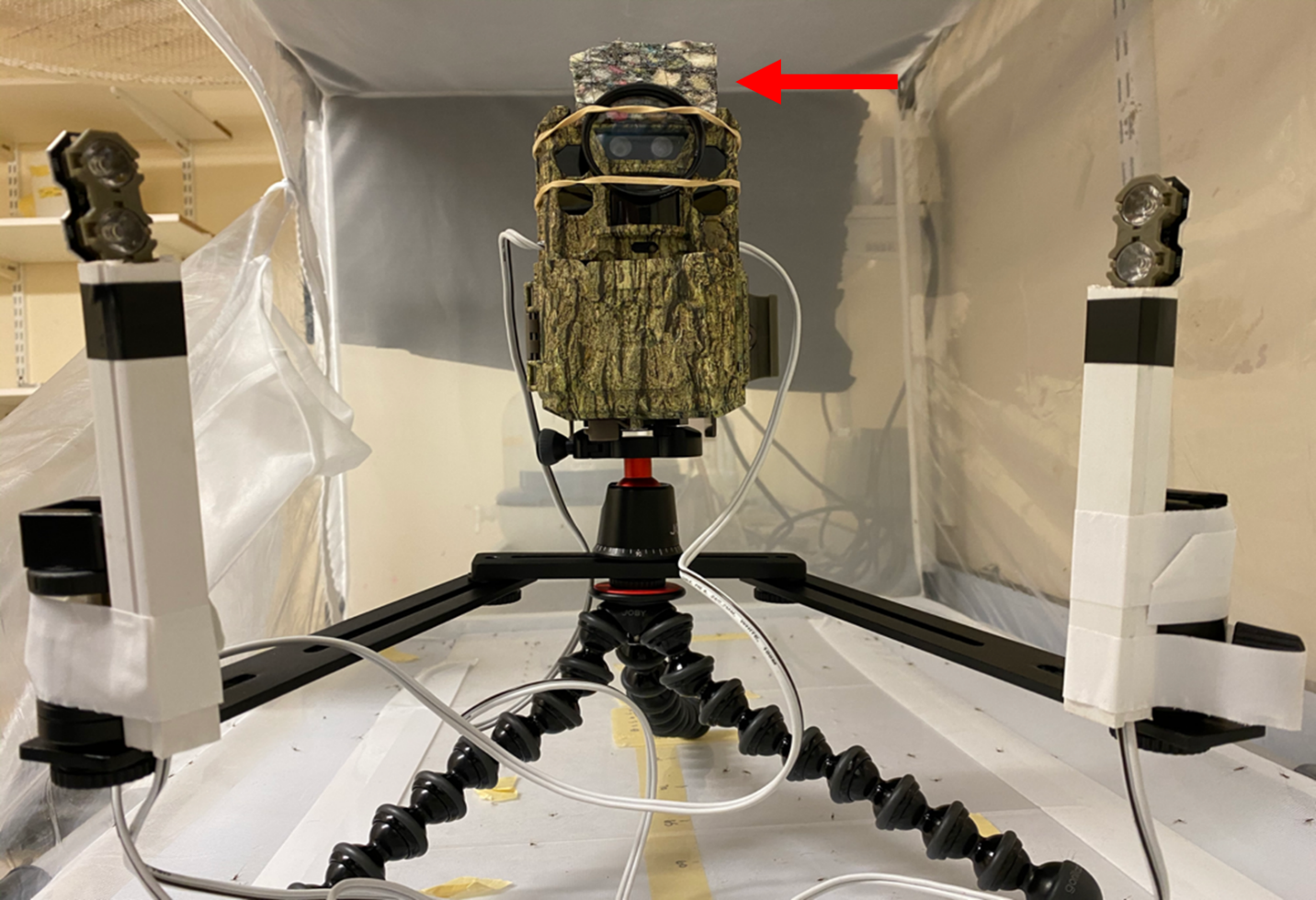
**

**Additional file 2. Fig. S2:** An adapter designed to hold a close-up filter in front of the dual lenses of the Bushnell CoreDS trail camera was developed for 3D printing in collaboration with the Keele School of Computing.

**
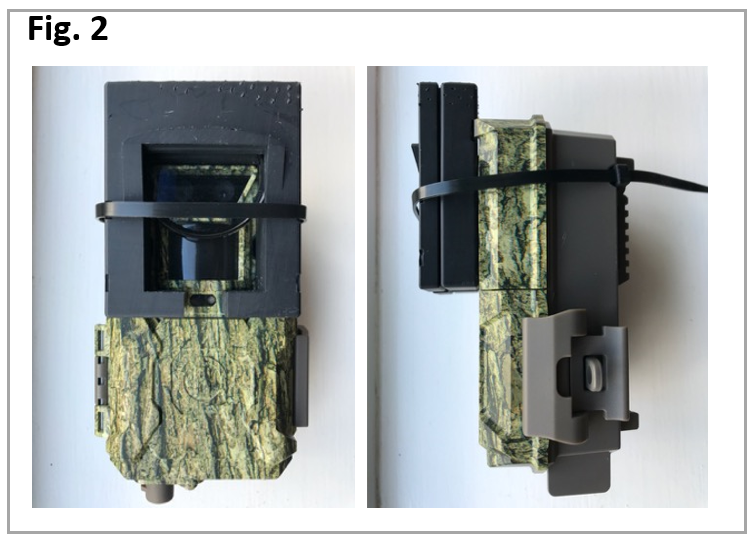
**

**Additional file 3: Text S1:** Operating instructions and optimal camera and LED infrared flash units’ position in relation to ASBs stations

**Trail Camera set-up for the monitoring of Mosquitoes landing on Attractive Sugar Bait**

**Materials:**

1. Bushnell Core DS 30MP No Glow Trail Camera
2. Lensbaby 46mm macro filter kit with +1, +2, and +4 dioptric strength filters
3. Joby GorillaPod 3K Pro Kit Tripod or equivalent
4. Modular camera mounting side brackets to hold the two re-wired LED infrared flashes
5. 32GB San Disk Extreme Pro SDHC card
6. Rechargeable Batteries (6 per camera).

**Method:**

1. Insert memory card (**32GB MAXIMUM**) into the card slot on the side of the camera, as well as batteries into the battery cartridge prior to turning the camera on.
2. Switch the camera into setup mode to begin programming the camera to the desired settings, remembering to press OK to confirm each setting after it has been input:
   1. **Mode:** CAMERA
   2. **Image Size:** 30MP
   3. **Flash Range:** LOW
   4. **Sensor Level (Controls the PIR sensor):** AUTO
   5. **Camera Mode:** 24HRS
   6. **Field Scan:** ON
      1. **Block A Start Time:** 1^st^ Desired Start Time
      2. **Block A Finish Time:** 1^st^ Desired End Time
      3. **Block B Start Time:** 2^nd^ Desired Start Time
      4. **Block B Finish Time:** 2^nd^ Desired End Time
   7. **Interval:** Dependant on desired length between photos
3. Once the required setting parameters have been input, the camera can now be switched to the ON position, to begin the capture procedure.
4. Mount the camera to the desired tripod or mount and attach the external LED infrared flash modules to the arms of the brackets at the distances and angles outlined.
5. Fix the **+2-macro filter** to the camera so that it covers both camera lenses using the 3D printed 2-piece adaptor and zip tie(s).
6. Camera is now ready to be placed at the optimal distance of **55cm** from the target bait station.

**Additional file 4. Fig. S3:** Draft foldable camera station design using aluminium hinged plates as frame and side wiring to prevent access to the camera.

**
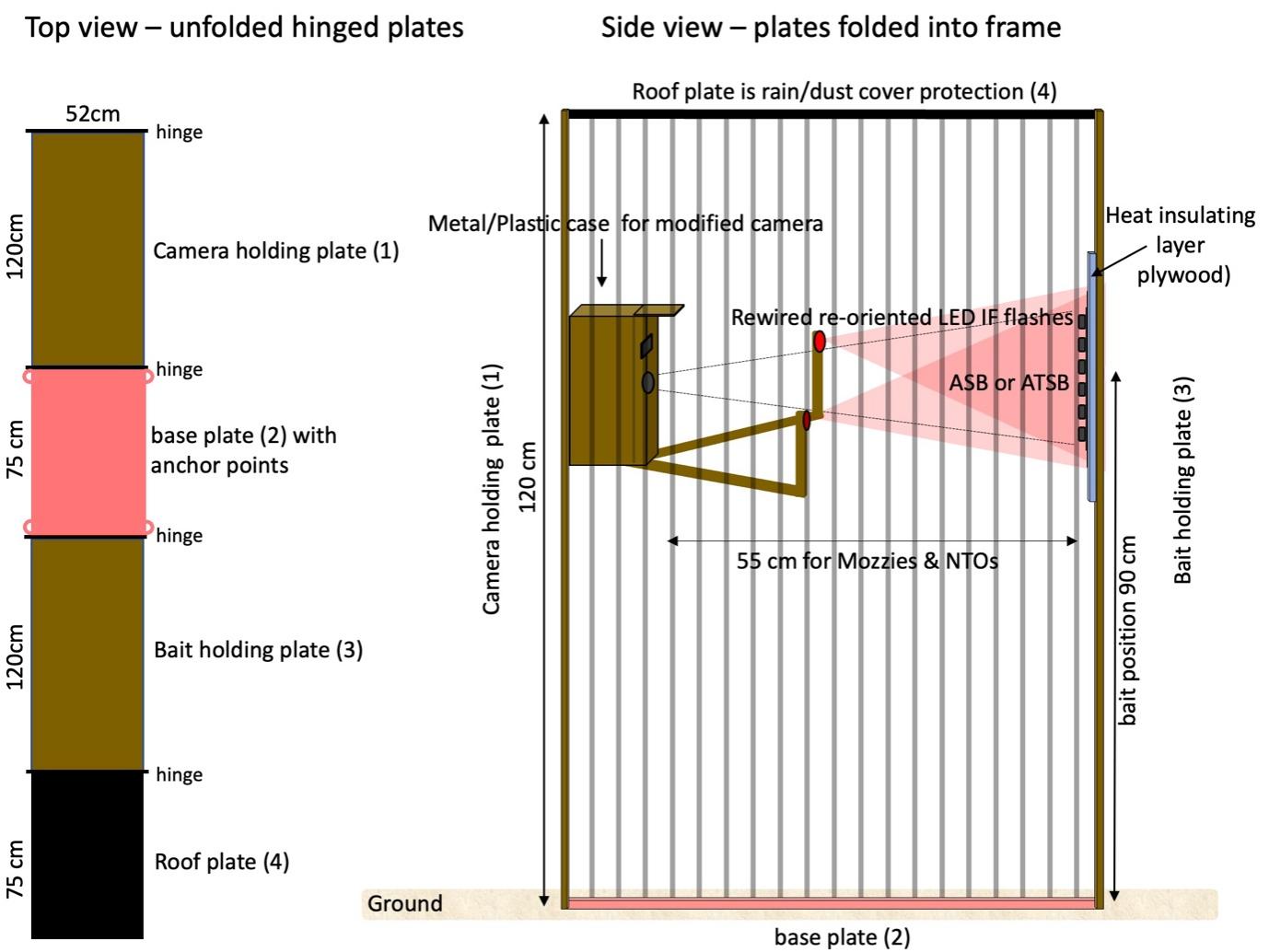
**

**Additional file 5. Fig. S4:** Images of the Westham ASB station with an attractant (version 1.0) taken within a large cage to optimize the system. Fluorescent tube light was used for "day light" optimization, and repositioned infrared flashes were used for night images.


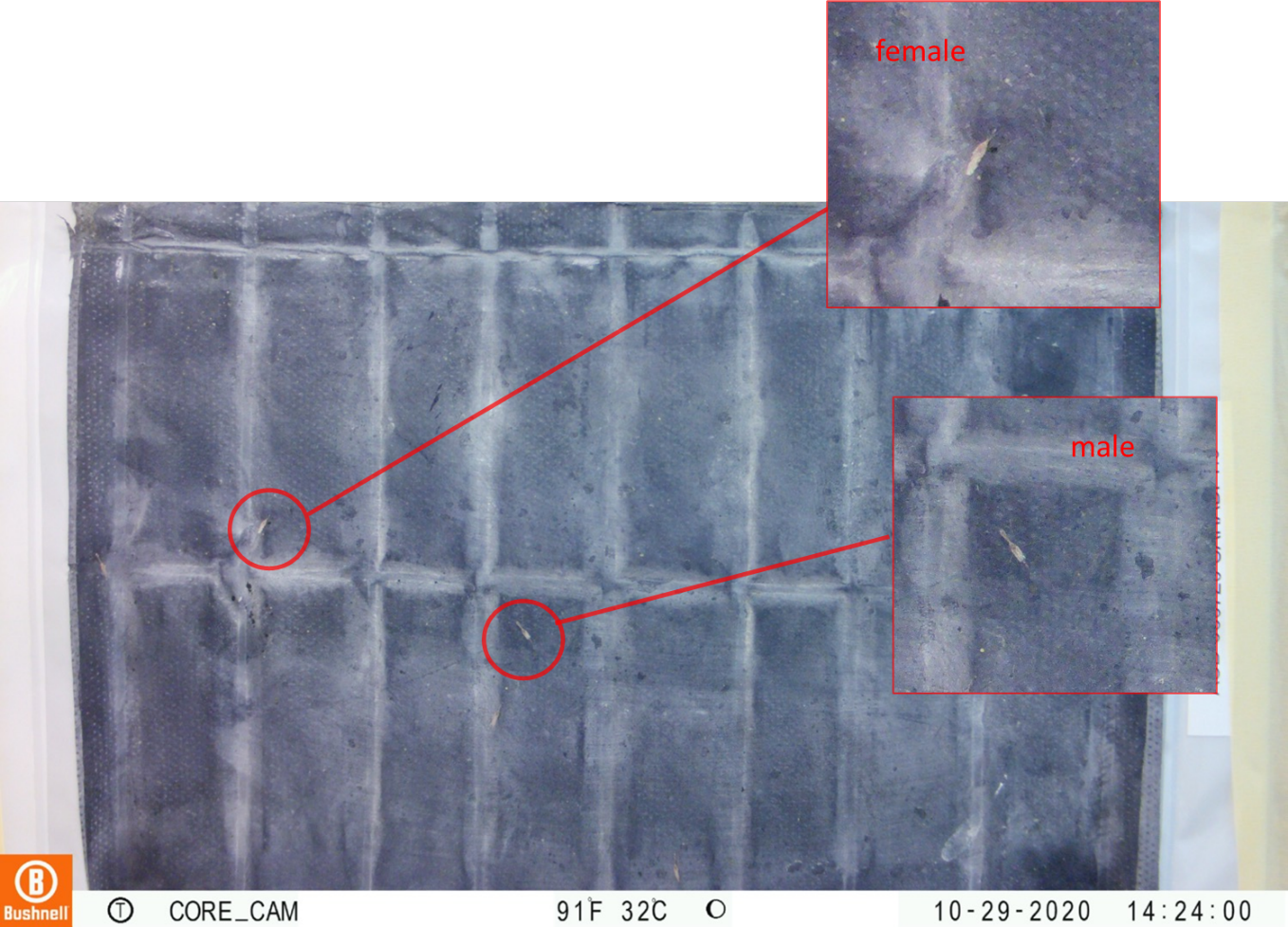


**Additional file 6. Fig. S5:** Recommended distances from the camera's objective (including close-up filter) to an ASB or ATSB surface and position and angle of the re-wired infrared flash.


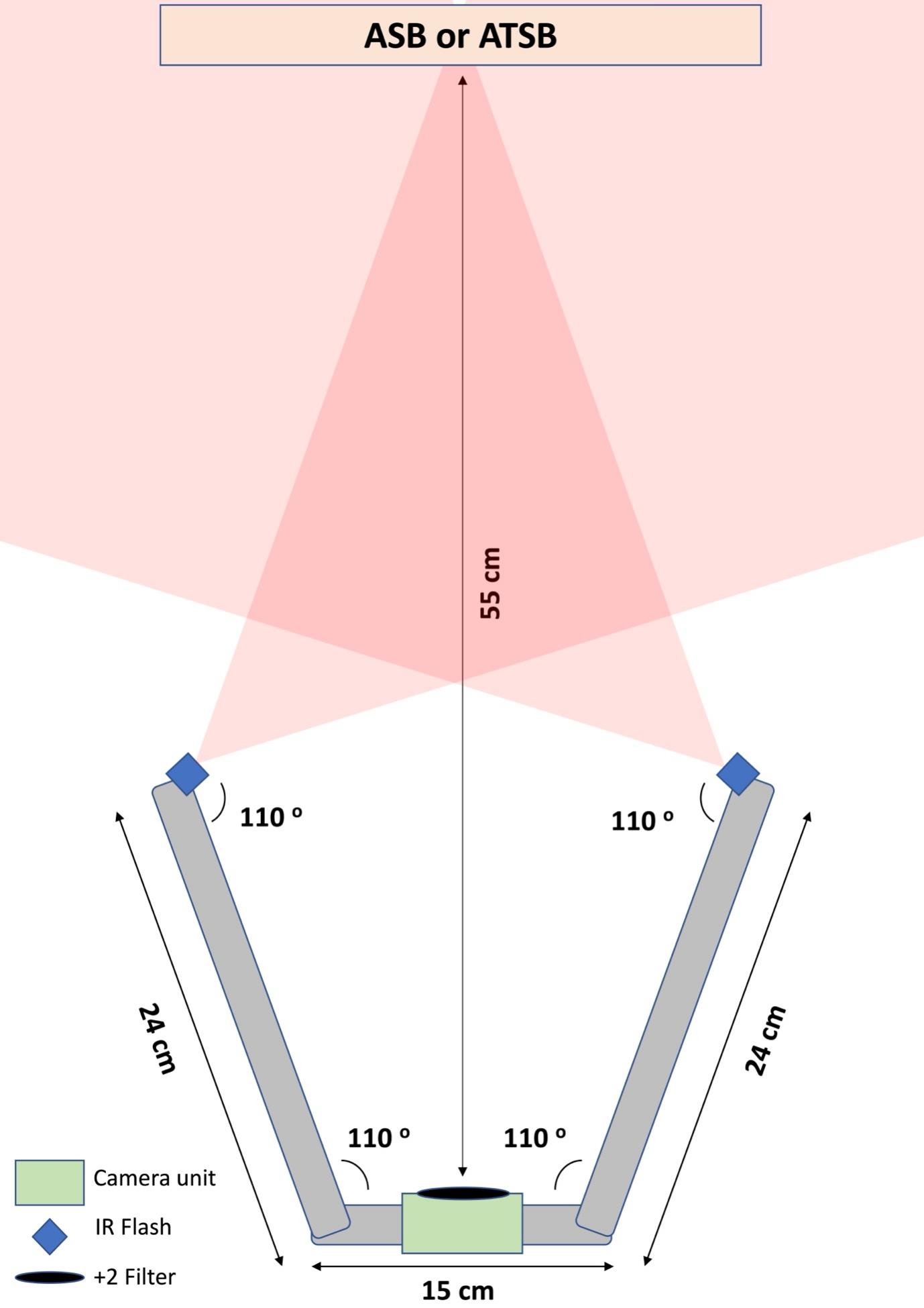


**Additional file 7. Fig. S6:** A map showing the positions of the camera traps in the field in Lupiro village, located in southeastern Tanzania.

**
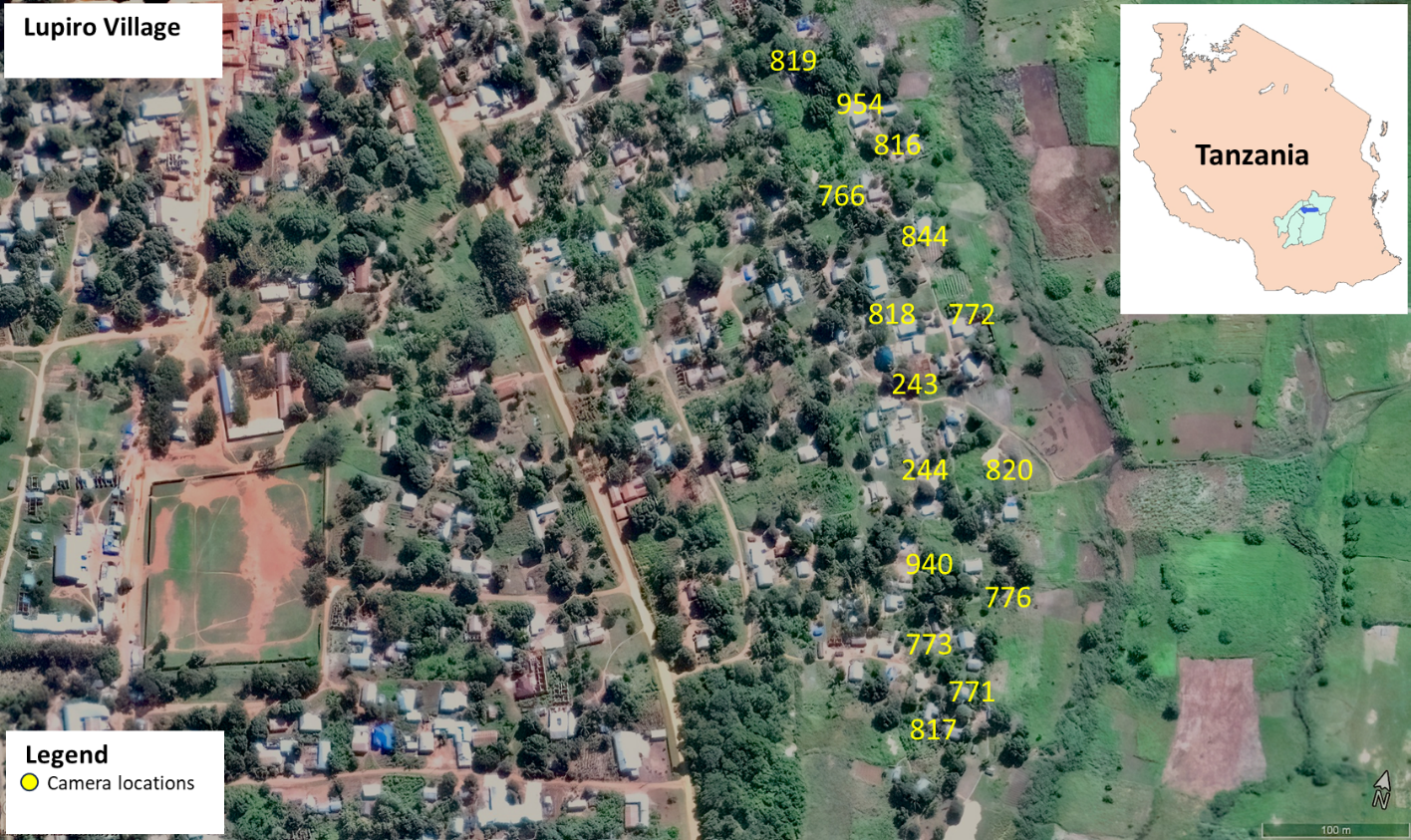
**

**Additional file 8. Fig. S7**: The camera station was designed with 4 aluminum plates locked into position, creating a rigid frame to support the camera safety box and bait holding plate and serving as the anchoring base and roof. The sides were made of heavy steel mesh, adding to the rigidity and sturdiness of the tamper-proof, animal-proof structure.


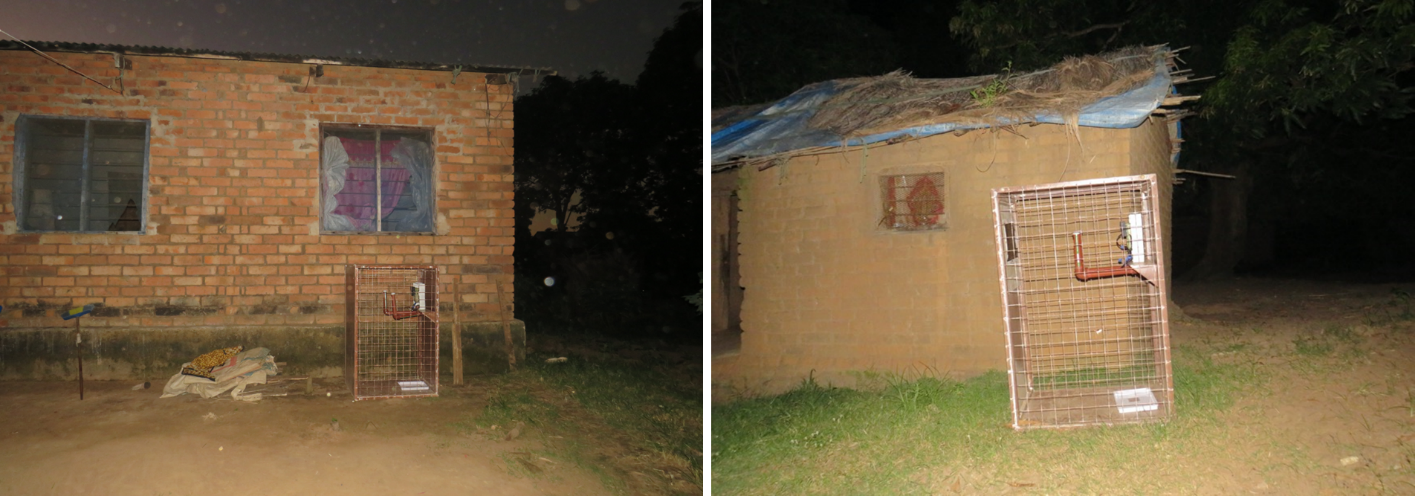


**Additional file 9. Fig. S8:** Outlier analyses of visit duration on baits (here >12 min) using logarithmic (top) and linear scales (bottom - distribution bounded to 1-100 min) - Boxplots indicate median and 1st and 3rd quartile, whilst whiskers extend to the 3^rd^ quartlile + 1.5*interquartile range. Datapoints beyond that limit were considered outliers (here >12 min) and indicative of mosquitoes resting or sleeping on the baits after their feeding bout.
